# Acute depletion of CTCF directly affects *MYC* regulation through loss of enhancer–promoter looping

**DOI:** 10.1093/nar/gkz462

**Published:** 2019-05-25

**Authors:** Judith Hyle, Yang Zhang, Shaela Wright, Beisi Xu, Ying Shao, John Easton, Liqing Tian, Ruopeng Feng, Peng Xu, Chunliang Li

**Affiliations:** 1 Department of Tumor Cell Biology, St. Jude Children’s Research Hospital, 262 Danny Thomas Place, Memphis, TN 38105, USA; 2 Howard Hughes Medical Institute, Chevy Chase, MD 20815-6789, USA; 3 Department of Computational Biology, St. Jude Children’s Research Hospital, 262 Danny Thomas Place, Memphis, TN 38105, USA; 4 Department of Hematology, St. Jude Children’s Research Hospital, 262 Danny Thomas Place, Memphis, TN 38105, USA

## Abstract

Numerous pieces of evidence support the complex, 3D spatial organization of the genome dictates gene expression. CTCF is essential to define topologically associated domain boundaries and to facilitate the formation of insulated chromatin loop structures. To understand CTCF’s direct role in global transcriptional regulation, we integrated the miniAID-mClover3 cassette to the endogenous *CTCF* locus in a human pediatric B-ALL cell line, SEM, and an immortal erythroid precursor cell line, HUDEP-2, to allow for acute depletion of CTCF protein by the auxin-inducible degron system. In SEM cells, CTCF loss notably disrupted intra-TAD loops and TAD integrity in concurrence with a reduction in CTCF-binding affinity, while showing no perturbation to nuclear compartment integrity. Strikingly, the overall effect of CTCF’s loss on transcription was minimal. Whole transcriptome analysis showed hundreds of genes differentially expressed in CTCF-depleted cells, among which *MYC* and a number of *MYC* target genes were specifically downregulated. Mechanically, acute depletion of CTCF disrupted the direct interaction between the *MYC* promoter and its distal enhancer cluster residing ∼1.8 Mb downstream. Notably, *MYC* expression was not profoundly affected upon CTCF loss in HUDEP-2 cells suggesting that CTCF could play a B-ALL cell line specific role in maintaining *MYC* expression.

## INTRODUCTION

CCCTC-binding factor (CTCF) is a transcription factor with 11 zinc finger domains ubiquitously expressed in cells of eukaryotes. Initially identified as a transcriptional repressor of chick *c-myc* (1,2), CTCF has since been shown to be a multi-functional gene necessary for survival and differentiation in mammals. Mouse genetic studies have revealed that *Ctcf* is required during early embryogenesis, with homozygous *Ctcf* knock-out mice survival limited to 5.5 days post coitum (dpc) (3,4). Although heterozygous *Ctcf* mice were viable and fertile, they exhibited a notably increased cancer incidence in aged animals (5). In addition, conditional knock-out mouse studies using *Ctcf*^flox/flox^ mice crossed with various tissue-specific Cre-expressing strains confirmed the requirement of *Ctcf* during organ development (6–9).

CTCF is an important regulator of the 3D chromatin architecture of interphase chromosomes, which guides gene expression (10–13). However, the complex and dynamic organization of the genome within the nucleus is not fully understood. At the broadest level of organization, chromosomes are spatially arranged into non-random chromosomal territories generally defined by nuclear localization, and these territories are organized at the multi-Megabase (Mb) scale into either open, active A compartments or closed, inactive B compartments (14–17). Compartment structures are further organized into various sized topologically associated domains (TADs), which provide an insulated neighborhood that maintains transcriptional regulation *in vivo* (18). CTCF is well-characterized as a transcription factor that binds to insulator elements to define TAD boundaries, and along with cohesin proteins, promote the formation of CTCF–CTCF insulated chromatin loop structures. These structures spatially compartmentalize interacting promoters and enhancers into regulatory domains that allow for dynamic gene transcriptional regulation through proximal and distal intra-TAD chromatin looping (11,18–20). Intra-TAD DNA loops between enhancer and promoter regions have also been shown to be facilitated by CTCF (18,21). Significantly, meta-analysis of CTCF-mediated chromatin loops identified the unique pattern that two CTCF-binding sites must be oriented in a convergent manner to favor direct DNA contact and binding by cohesin proteins (21).

Previous attempts to address CTCF’s role in genome organization and transcriptional regulation in human cell lines have relied on either targeted disruption of CTCF-binding sites at specific TAD boundaries (22–24) or global RNA depletion by siRNA and antisense oligos (25,26), none of which are reversible. While these studies offered valuable insights into CTCF’s involvement in TAD boundary insulation and chromatin looping maintenance, the studies were not sensitive enough to fully deplete CTCF in an acute manner. In addition, accumulated secondary transcriptional responses might occur during the long-term establishment of knockdown cell lines. More recently, the auxin-induced degron (AID) system was utilized in a mouse embryonic stem (ES) cell model to integrate a miniAID epitope tag fused to the mClover3 fluorescent reporter gene to the endogenous *Ctcf* locus by clustered regularly interspaced short palindromic repeats (CRISPR/Cas9) genome editing. In the presence of doxycycline and auxin, enforced expression of OsTIR1 combines with Skp1/Culin/F-box (SCF) ubiquitin ligase components in the cell to form a functional SCF/OsTIR1 E3 ubiquitin ligase complex that rapidly degrades Ctcf protein in minutes, which was reversible after doxycycline and auxin were completely removed (27–29). Results from the study showed that loss of Ctcf did not abrogate genomic organization at the larger, compartmental scale, but did disrupt TAD insulation and chromatin looping between boundaries, resulting in a 10-fold increase in differentially expressed genes over a 4-day time course. Compared with previous functional studies of CTCF utilizing mRNA knockdown by shRNA, antisense oligo or Cre/LoxP induced *Ctcf*^+/−^ tissues (4,5,30), AID system allowed for complete depletion of Ctcf in an acute and reversible manner, and greatly improved the investigation to identify Ctcf’s direct effect on chromatin organization and transcriptional regulation.

In human cells, CTCF’s role in genomic organization and global transcription regulation is less clearly defined due to the lack of available tools to efficiently deplete CTCF. Here, we used an AID system to conditionally deplete CTCF in a human B-cell lymphoblastic leukemia (B-ALL) cell line, SEM (31), and an immortal human erythroid precursor cell line, HUDEP-2 (32). To create the reporter cell lines, we developed a novel knock-in strategy termed CHASE-knock-in that combined homology-mediated end joining (HMEJ)-based (33) donor design with flow cytometry based serial sorting to improve knock-in efficiency in hard to target human cell lines. Our data supported the recent reported data that acute loss of Ctcf does not affect nuclear compartmentalization on the multi-Mb scale, but does significantly disrupt TAD boundary integrity and enhancer–promoter looping (29,34). Notably, even though chromatin organization was altered, the impact on global transcription was minimal suggesting the direct targets of CTCF were limited. Longer depletion of CTCF may be necessary for chromatin architectural changes to affect gene regulation of indirect targets. Here, we present evidence supporting that *MYC* was highly susceptible to CTCF depletion, and that the transcriptional regulation of *MYC* was mediated through a long-distance CTCF dependent enhancer–promoter interaction.

## MATERIALS AND METHODS

### Vector construction

The all-in-one CRISPR/Cas9-CTCF-gRNA (V6) construct was derived from a previous study (35). All target-specific single guide RNAs (gRNAs) were predicted by online software (http://crispr.mit.edu/) (36). Paired oligomers containing 20 bp of selected target sequence were synthesized from Thermo Fisher Scientific Company and cloned into the all-in-one vector between BsmBI sites. All oligo information were listed in Supplementary Table S1. Individual bacterial clones were screened and confirmed by Sanger sequencing with the U6-Forward sequencing primer: 5′GGGCAGGAAGAGGGCCTAT3′. A two-step in-fusion cloning protocol was performed to generate the CTCF-miniAID-mClover3 donor knock-in vector. Cloning primers were designed to amplify the 800 bp HA 5′ to the endogenous CTCF gRNA target. Overhangs of 23 bp including the target gRNA and PAM sequences (5′GCACAAGGCTCCGCCATCACCGG3′) were added to the 5′ end of the forward primer amplifying the 5′ HA sequence. The 3′ HA was generated with a similar design (reverse primer containing 23 bp including the target gRNA and PAM sequences), but also included an additional overhang for in-fusion cloning (Clontech). Both HAs were amplified from genomic DNA of SEM cells. The miniAID-mClover3 DNA fragment was amplified from pMK290 vector (Plasmid #72828, Addgene) (37). Snapgene software was used to design all primers used for in-fusion cloning. The PCR reactions were performed using CloneAmp polymerase (Clontech) and the cycling parameters were as follows for all cloning: 98°C for 5 min, followed by 40 cycles of 98°C for 15 s, 55°C for 20 s and 72°C for 20 s. First, the amplified 5′ HA was cloned into pCR-Blunt II-TOPO vector (Thermo Fisher Scientific). The DNA was purified from colonies and screened by Sanger sequencing with the primers M13F and M13R. The pCR-Blunt II-TOPO-gRNA-PAM-5′HA was then linearized by PacI digestion and ligated with miniAID-mClover3, and the 3′HA-gRNA-PAM through in-fusion cloning protocol (Clontech). Sanger sequencing was performed to ensure that the knock-in DNA was seamlessly cloned in-frame with the CTCF peptide. The doxycycline-regulated OsTIR1 expression plasmid (pMK243) was purchased from Addgene (Plasmid #72835, Addgene). The primers and sequences used for cloning vectors are listed in Supplementary Table S1.

### Cell culture

The human B-ALL cell line SEM (DSMZ) was maintained in RPMI-1640 medium (Lonza) containing 10% fetal bovine serum (FBS) (Hyclone), 2 mM glutamine (Sigma) and 1% penicillin/streptomycin (Thermo Fisher Scientific). Immortal human erythroid precursor cell line HUDEP-2 was expanded in StemSpan SFEM (Stem Cell Technologies) supplemented with 1 μM dexamethasone, 1 μg/ml doxycycline, 50 ng/ml human SCF, 3 Units/ml EPO and 1% penicillin/streptomycin. All cells were maintained at 37°C in a 5% CO_2_ atmosphere and 95% humidity. All cells were tested negative for mycoplasma infection. Cell identity of SEM was confirmed by short tandem repeat (STR) analysis.

### Electroporation

SEM and HUDEP-2 cells were electroporated by using the Nucleofector-2b device (Lonza) with the V-kit and program X-001. For CTCF-miniAID-mClover3 knock-in delivery, 2.5 μg of the donor plasmid and 2.5 μg of the CRISPR/Cas9-CTCF-gRNA plasmid were used for 5 million SEM cells. For the doxycycline-regulated OsTIR1 integration, 2.5 μg of pMK243 and 2.5 μg of the CRISPR/Cas9-AAVS1-gRNA plasmid were co-delivered into 5 million SEM cells.

### Flow cytometry

To determine the percentage of cells that were mClover3-positive, suspension cultured SEM cells were collected by centrifugation at 800 *g* and filtered through a 70 μm cell filter before flow cytometry sorting. Fluorescence from mClover3 was detected by using the same FL1/FITC channel as GFP. DAPI was added to the cell suspension to exclude dead cells.

### Characterization of on-target knock-in events by genomic PCR and Sanger sequencing

All cells stably integrated with knock-in cassettes were harvested for DNA extraction by PureLink Genomic DNA Mini Kit (Thermo Fisher Scientific) 5 weeks after co-electroporation with the CTCF-miniAID-mClover3 donor vector and CRISPR/Cas9-CTCF-gRNA plasmid. Specific primer sets designed to recognize the 5′ and 3′ knock-in boundaries were used with the following PCR cycling condition: 98°C for 5 min, followed by 40 cycles of 98°C for 15 s, 55°C for 20 s and 72°C for 20 s. The bands with expected size were cut out, purified and ligated to pCR-Blunt II-TOPO vector for Sanger sequencing by the M13F and M13R primers. Single cell-derived clones 27, 35 and 42 were genotyped for homozygous insertion of the miniAID-mClover3 cassette, and clone 36 for heterozygous knock-in.

### Immunoblotting

Cells lysate was prepared by using RIPA buffer followed with SDS-PAGE (Thermo Fisher Scientific) and transferred to a PVDF membrane according to the manufacturer’s protocols (Bio-Rad) at constant 100 V for 1 h. After blocking incubation with 5% non-fat milk in TBS-T (10 mM Tris, pH 8.0, 150 mM NaCl, 0.5% Tween-20) for 1 h at room temperature, the membrane was incubated with antibodies against GAPDH (Thermo Fisher Scientific, AM4300, 1:10,000), AID (MBL, M3-214-3, 1:2,000), MYC (Cell Signaling Technology, #9402, 1:1,000), ACTIN (Sigma, MABT825, 1:5,000) and CTCF (abcam, ab70303, 1:1,000) at 4°C for 12 h with gentle shaking. Membranes were washed three times for 30 min and incubated with a 1:2,000 dilution of horseradish peroxidase-conjugated anti-mouse or anti-rabbit antibodies for 2 h at room temperature. Blots were washed with TBS-T three times for 30 min and developed with the ECL system (Amersham Biosciences) according to the manufacturer’s protocol.

### Auxin-induced degradation

Successful knock-in clones were cultured in medium supplemented with doxycycline (1 μg/ml) to induce the expression of OsTIR1. Three single-cell derived clones were treated with culture medium supplemented with 500 μM IAA (natural auxin) (Sigma) for 24 or 48 h to induce degradation of CTCF. ‘Wash’ cells were prepared from treated cells that were centrifuged and resuspended by PBS three times followed with an additional culture for 48 h in regular medium.

### RNA-sequencing

Total RNA was extracted by Trizol (Thermo Fisher Scientific, 15596026) from replicate samples of cells treated with or without IAA for 24 or 48 h. About 200 ng total RNA were treated using Kapa rRNA depletion reagents to remove ribosomal RNA, then converted into cDNA libraries using Kapa RNA HyperPrep Kit with RiboErase (HMR). After end repair, dA-tailing and adapter ligation, each cDNA library was purified and enriched by 11 cycles of PCR amplification. All RNA-seq libraries underwent 151-cycle paired-end sequencing on the Illumina NextSeq 500 system. FASTQ data of RNA-Seq were mapped to genome hg19. RNA-Seq reads from each species were aligned using Bowtie2 2.3.4 (38). Fragments per kilobase per million sequenced reads (FPKM) value were obtained using cufflinks v2.2.1 (39). Per-gene read counts were obtained using HTSeq v. 0.6.1 (40). Differentially expressed genes (DEGs) were identified using count data generated as described above and DESeq2 (version 1.14.0) (41). The log_2_(Fold change) of all genes from whole transcriptome comparison (CTCF^AID-IAA^ versus CTCF^AID+IAA^) were uploaded for GSEA analysis (42). FPKM values of all genes were provided in Supplementary Table S2.

### Quantitative real-time PCR

Reverse transcription was performed by using the High-Capacity cDNA Reverse Transcriptase Kit (Applied Biosystems, 4374966). Real-time PCR was performed using FAST SYBR Green Master Mix (Applied Biosystems, 4385612) based on manufacturer’s instructions with primers to detect *CTCF, MYC* and *GAPDH* (Supplementary Table S1). Relative gene expression was determined by using the ΔΔCT method (43).

### Hi-C and data analysis

*In situ* Hi-C experiments were carried out as previously described (18). Briefly, five million CTCF^AID^ SEM cells (clones 27 and 35) with or without IAA treatment were crosslinked with 1% formaldehyde for 10 min at room temperature, digested with 125 Units of MboI, labeled by biotinylated nucleotides and ligated for proximity ligation. After de-crosslinking, ligated DNA was purified and sheared to 300–500 bps. Ligation junctions were then pulled down with streptavidin beads and prepped as a standard Illumina library. Each library underwent 75-cycle paired-end sequencing on Illumina HiSeq 4000 system. About 100 million reads were obtained for each sample. Raw sequence data were mapped and processed using Juicer v1.5 (44) with default parameters. The Hi-C data and MboI cut sites were mapped to hg19. Replicates data were first processed separately. After confirmation of good reproducibility by HiC-Spector (Supplementary Figure S3C) (45), we merged the replicates and re-processed as combined results. The data were visualized with the Juicebox (46). Our Hi-C samples’ resolution was between 8 and 20 kb, and the median resolution of merged samples was ∼18.5 kb. TADs and loops were called by Arrowhead and HiCCUPS from Juicer pipeline. We provide their annotated results in Supplementary Table S3. The Cis Eigenvector values were calculated by eigenvector function of Juicebox at 250 kb resolution.

### Capture-C

Capture-C was performed on two CTCF^AID^ knock-in clones 27, 35 and the CTCF^AID^ heterozygous knock-in clone 36 following the protocol described by Davies JO *et al.* (47). To quantify interaction frequency between *MYC* promoter and distal enhancers, signal value from .bw file was analyzed by bigWigToBedGraph, then normalized by probe signal for each experiment (48). Capture-C oligo information was included in Supplementary Table S1.

### Cut&Run assay

Cut&Run assay of CTCF was performed as previously described (44). Three million cells were collected for each sample. Cells were pelleted at 600 *g* for 3 min at room temperature. Cell pellets were washed twice with 1.5 ml room temperature wash buffer. Cell pellets were resuspended in 1 ml wash buffer at room temperature. While gently mixing, 100 μl concanavalin beads (BioMagAPlus Concanavalin A, Polysciences, 86057-3; prewashed and resuspended in 100 μl binding buffer) were added to the samples, followed with gentle rotation for 10 min at room temperature. Samples were placed on a magnetic stand and the supernatant was removed. Beads were resuspended in 200 μl AB buffer (Dig-wash buffer with 0.02% digitonin and 0.5 M EDTA) with 1.6 μg CTCF antibody (abcam, ab70303). Samples were rotated at 4°C for 2 h. Samples were placed on a magnetic stand and supernatant was removed. Beads were gently resuspended in 200 μl Dig-wash buffer with 0.02% digitonin, and 2 μl PA-MN (homemade reagent kindly provided by Dr Steven Henikoff) was added to each sample, followed by rotation at 4°C for 1 h. Samples were placed on a magnetic stand and supernatant was removed. Beads were washed for a total of three times in 1 ml Dig-wash buffer, pipetting gently. Following the final wash, beads were resuspended in 100 μl Dig-wash buffer by gentle mixing and transferred to a new tube. On ice, 2 μl 100 mM CaCl_2_ was added to the sample with gentle mixing. Afterward, samples were incubated on ice for 30 min. Then 100 μl stop buffer was added to the samples and samples were incubated for 10 min at 37°C followed by centrifugation at 4°C, 12,000 rpm. Samples were placed on a magnetic stand and the supernatant containing the Cut&Run fragments was collected. To the samples, 2 μl 10% SDS and 2.5 μl proteinase K were added and the samples were incubated at 50°C overnight. DNA was extracted by the phenol/chloroform/isopropanol protocol. Library construction was performed using the NEBNext UltraII DNA Library Prep Kit from NEB (E7645S). Indexed samples were sequenced using the Illumina MiSeq V3 600-cycle kit (MS-102-3003).

### Analysis of Cut&Run data

Paired-end reads were trimmed for NEB index adapter by cutadapt (version 1.9, paired-end mode, default parameter with ‘-m 6 -O 20’) and aligned to human genome hg19 by BWA (version 0.7.12-r1039, default parameter) (49). Duplicated reads were then marked with Picard (version 2.6.0-SNAPSHOT) and only non-duplicated proper paired reads were kept by samtools (parameter ‘-q 1 -F 1804’ version 1.2) (50). To evaluate the Cut&Run assay quality, we first separated reads by fragment size <120 bp, or fragment size >150 bp and <2,000 bp and generated bigwig files for visualization. We observed a clear pattern that peaks in tracks with <120 bp were sharper and surrounded by peaks in tracks with >150 bp on IGV (version 2.4.13), which was consistent to the description from the original Cut&Run protocol paper (51). We then generated bigwig files using all reads that fragment size <2,000 bp. Our samples contained 4–11 million uniquely mapped reads that are comparable to published data. A R package ChIPseeker (version 1.18.0) was used for annotating Narrow peaks from MACS2 (52,53).

To check whether our reduced CTCF sites upon CTCF loss demonstrated bias toward 2XCTSes compared with CTCF only sites (1XCTSes), we first called peaks individually from each of the three clones (clones 27, 35 and 42 with or without IAA) by MACS2 (version 2.1.1.20160309, ‘-f BAMPE –extsize 200 –nomodel’) twice using high confidence cutoff FDR correct *P*-value ≤0.05; or low confidence cutoff FDR correct *P*-value ≤0.5, respectively. To obtain reproducible peaks, we only kept peaks called at least once in high confidence cutoff and in either high or low confidence cutoff for the other two replicates. After merging reproducible peaks from samples with or without IAA and removing overlapped peaks (blacklist) (54), we counted reads from three single cell derived clones with overlapped peaks, calculated FPKM and compared between +IAA versus -IAA groups. To further define CTCF peaks with high stringency, we scanned genome-wide CTCF motif predicted by TRANSFAC (55) and JASPAR (56) using FIMO from MEME suite (57) (version 4.11.3, ‘–motif-pseudo 0.0001 –thresh 1e-4’) and kept peaks carrying CTSes annotated by ‘TRANSFAC_CTCF_M01259’ (Figure 2D-E and Supplementary Figure S2H). The ratio calculated by log_2_fold change (CTCF^AID+IAA/-IAA^) was utilized to define reduced (log_2_fold change≤-1), retained (-1<log_2_fold change≤1) and gained (log_2_ fold change>1) CTSes.

### Statistical analysis

Statistical analysis was performed by GraphPad Prism 6.0. *P*-values in Q-PCR, RNA-seq and quantification of Capture-C results were calculated by performing a two-tailed *t*-test from two or three independent biological replicates.

## RESULTS

### The miniAID cassette knock-in to the endogenous CTCF locus

**Figure 1.**
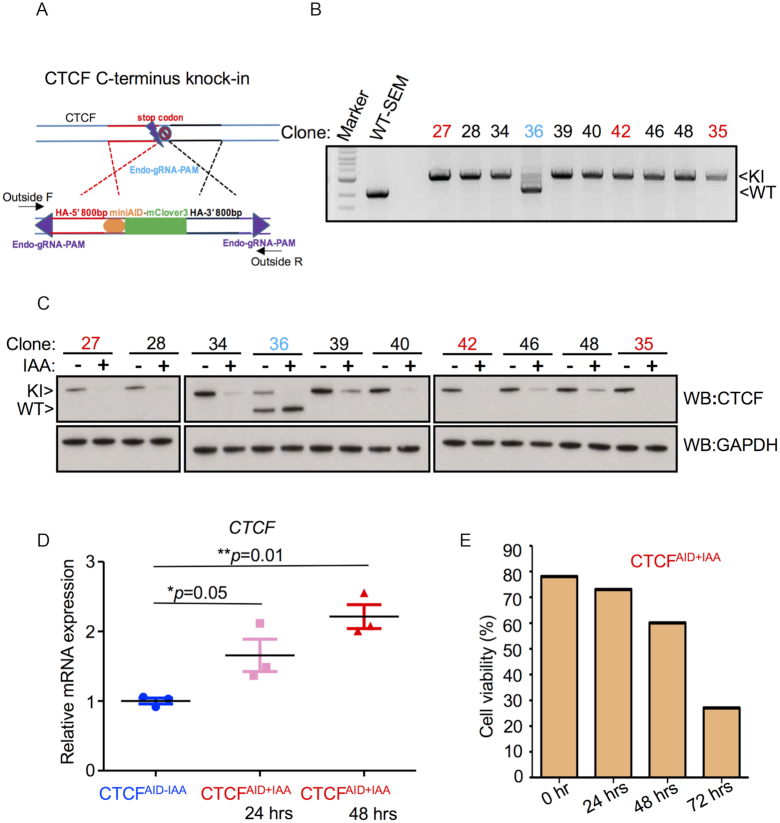
miniAID cassette knock-in to the endogenous *CTCF* locus. (**A**) Schematic diagram of the donor vector design for miniAID-mClover3 knock-in to the C-terminus of human *CTCF* before the stop codon. HA: homology arm; Endo-gRNA-PAM: CTCF endogenous guide RNA sequence and PAM sequences added to each end of the HAs. (**B**) A pair of primers residing outside the HAs was used for genomic PCR. Single-cell derived clones (Clones 27, 28, 34, 35, 39, 40, 42, 46 and 48) were identified to only carry the knock-in (KI) allele. Clone 36 was recognized as a mono-allelic knock-in clone (labeled blue). Clones 27, 35 and 42 were selected for subsequent studies (labeled red). (**C**) Multiple single cell-derived clones were expanded and treated with IAA and doxycycline for 48 h to degrade the CTCF-miniAID-mClover3 fusion protein. Knock-in clones 27, 35 and 42 demonstrated complete protein degradation after IAA treatment, as detected by immunoblotting. Mono-allelic knock-in clone 36 demonstrated specific degradation of the fusion protein translated from the knock-in allele and retained endogenous protein expression. The fusion protein was 35 KD larger than endogenous CTCF. GAPDH was included as loading control. (**D**) Q-PCR assessment of *CTCF* mRNA in response to CTCF protein depletion from three biological replicates treated with IAA for 48 h; knock-in clones 27, 35 and 42 (*N* = 3). (**E**) Clone 27 cells were treated with doxycycline and IAA for 0, 24, 48 and 72 h and assessed for viability with trypan blue staining.

We developed an optimized CRISPR/Cas9 with homology arm and sorting enrichment knock-in protocol, so called CHASE-knock-in, which combined HMEJ donor design (33) with flow cytometry based serial sorting to efficiently deliver the miniAID-mClover3 cassette to the endogenous *CTCF* locus in a human B-ALL cell line, SEM (31), and an immortal human erythroid precursor cell line, HUDEP-2 (32), each with doxycycline inducible OsTIR1 integration (Figure 1A and Supplementary Figure S1A). Briefly, CHASE-knock-in requires co-delivering to cells the previously described CRISPR/Cas9 all-in-one vector (58) along with a knock-in cassette vector to generate CRISPR mediated double-cut nuclease cleavage and release of the knock-in cassette along with endogenous gRNA targeting to facilitate knock-in events, which were enriched by serial sorting for knock-in cassette fluorescence. In this study, a gRNA targeting the stop codon of *CTCF* was cloned into the CRISPR/Cas9 all-in-one vector, called CRISPR/Cas9-CTCF-gRNA. The knock-in cassette vector, called CTCF-miniAID-mClover3, was designed to flank a miniAID-mClover3 cassette with homology arms (HAs) 800-bp upstream (5′ HA) and 800-bp downstream (3′ HA) from the *CTCF* gRNA target sequence. The ends of the HAs in the knock-in cassette were equipped with the *CTCF* gRNA and PAM sequence, which allowed for the knock-in cassette to be released from the vector in the presence of CRISPR/Cas9-CTCF-gRNA (Figure 1A). Twenty-four hours after co-delivery of CRISPR/Cas9-CTCF-gRNA plasmid and CTCF-miniAID-mClover3 donor vector by electroporation, cells were sorted for fluorescence of CRISPR/Cas9-CTCF-gRNA (mCherry). The sorted population recovered for 3 weeks in culture, followed by a second sort to detect the proportion of cells expressing the knock-in reporter fluorescence, mClover3. In SEM cells, we detected ∼0.1% of the cells carried the mClover3 knock-in cassette following the second sorting. The cells containing the knock-in reporter were cultured for an additional 2 weeks and sorted a third time to expand the knock-in population. While initial knock-in efficiency was low, serial sorting led to an enriched knock-in population with 98.9% of the cells containing the mClover3 cassette following the third sort (Supplementary Figure S1B–D). High fluorescence density populations were selected to confirm successful knock-in events (Supplementary Figure S1E).

Following CHASE-knock-in, clones derived from a targeted bulk population of cells exhibiting high mClover3 levels were characterized for successful knock-in integration by genomic PCR and validated by Sanger Sequencing (Figure 1B). Auxin-inducible protein degradation was possible when *CTCF* was tagged with miniAID-mClover3 in SEM and HUDEP-2 cells with doxycycline inducible OsTIR1 integration. To test the efficient depletion of the CTCF-miniAID-mClover3 fusion protein, multiple knock-in clones were exposed to IAA (auxin) and doxycycline for 48 h in culture, and then harvested for immunoblotting using antibodies against CTCF and AID. Protein degradation was consistent between immunoblotting and flow cytometric analysis (Figure 1C; Supplementary Figure S1F–G). Parental wild-type SEM cells demonstrated no change to CTCF’s expression when treated with IAA and doxycycline (Supplementary Figure S1H). Thereafter, three knock-in SEM clones (27, 35 and 42) showing complete depletion of CTCF were selected for subsequent experiments. Additionally, three knock-in HUDEP-2 clones (C3, C16 and C41; Supplementary Figure S8A–D) were characterized and used to compare findings observed from experiments in the SEM clones 27, 35 and 42. In the mono-allelic knock-in SEM clone 36, when the CTCF-miniAID-mClover3 was degraded, CTCF protein translation from the wild-type allele notably increased, suggesting compensation occurred in response to CTCF disruption. We also investigated the mRNA expression of *CTCF* in the knock-in SEM clones 27, 35 and 42 by Q-PCR. After CTCF protein depletion, the mRNA level was significantly induced suggesting that CTCF protein might repress its own transcription (Figure 1D). Consistent with previous observations in mouse embryonic stem cells (33), loss of CTCF in SEM cells resulted in time-dependent cell death (Figure 1E) limiting the length of time cells could be treated and studied. Therefore, our human CTCF^AID^ cellular model was used as a tool to investigate early changes to genomic organization and global transcriptional regulation upon CTCF loss.

### CTCF degradation disrupted global CTCF binding affinity

**Figure 2.**
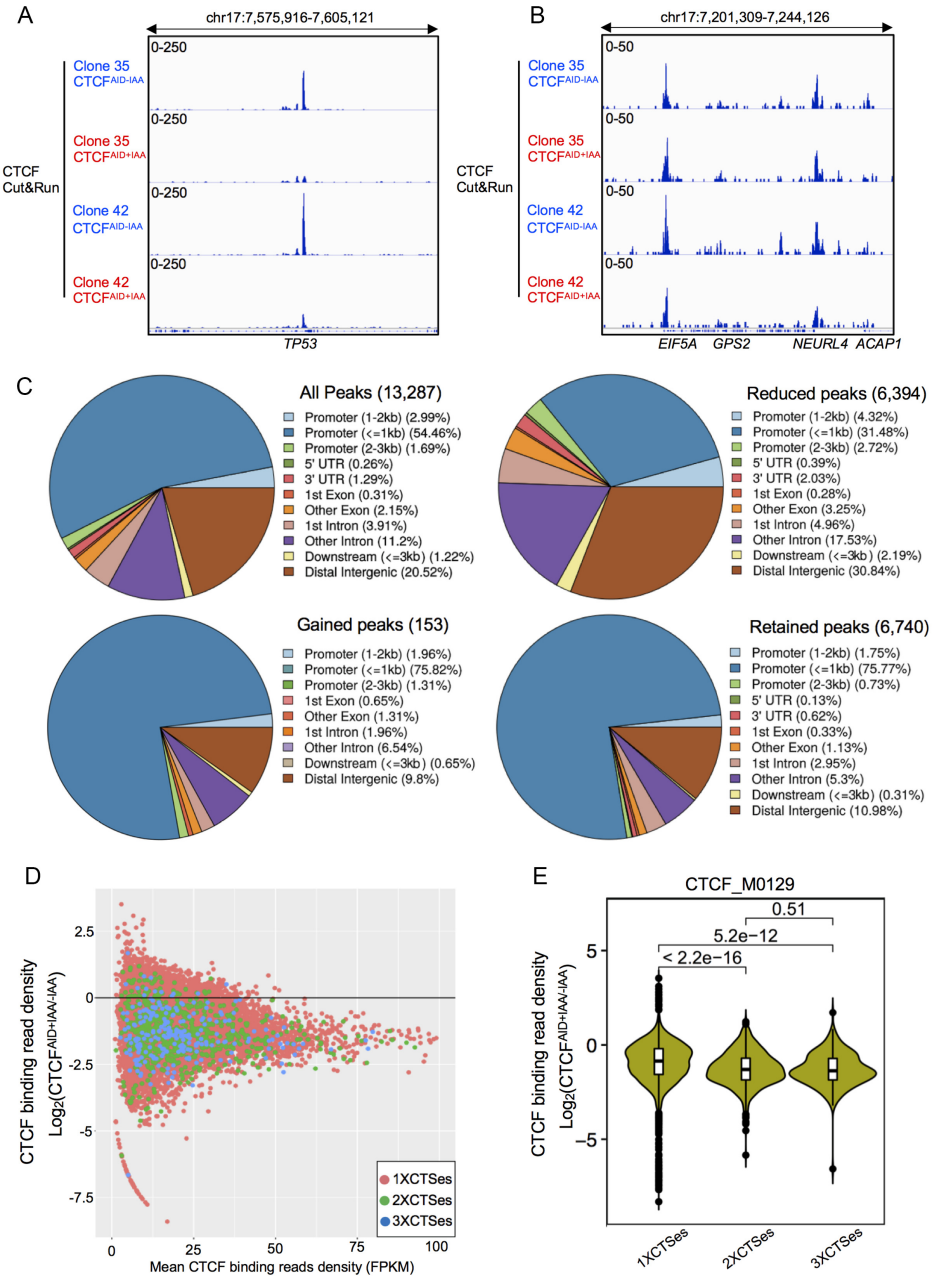
CTCF degradation disrupted global CTCF occupancy. (**A**) CTCF Cut&Run tracks shown at the selective viewpoint of *TP53* locus where significant reduction of CTCF binding following 48 h IAA treatment was observed from clones 35 and 42. (**B**) CTCF Cut&Run tracks shown at the selective viewpoint of *EIF5A* locus where minimal reduction of CTCF binding following 48 h IAA treatment was observed from clones 35 and 42. (**C**) Genomic distribution of all CTCF binding peaks carrying CTSes were analyzed by ChIPseeker for all peaks (13,287), reduced peaks (6,394), retained peaks (6,740) and gained peaks (153) collected from clones 27, 35 and 42 with and without IAA treatment for 48 h. (**D**) Global reduction of CTCF binding affinity was demonstrated by MA plot for 1XCTSes, 2XCTSes and 3XCTSes. The *X*-axis indicated mean CTCF binding read density calculated by FPKM from Cut&Run analysis, while *Y*-axis indicated log_2_fold change (+IAA/-IAA) of CTCF binding read density. (**E**) Statistical analysis of CTCF binding reduction among 1XCTSes, 2XCTSes and 3XCTSes groups was performed by showing Wilcox *P* value.

To assess the genome-wide CTCF-binding profile in CTCF depleted cells, we investigated CTCF binding occupancy using *in situ* chromatin immunoprecipitation followed by high-throughput sequencing (Cut&Run assay) (51). We identified 15,583, 33,059 and 21,669 peaks occupied by CTCF in clones 27, 35 and 42 without IAA treatment, respectively (Supplementary Figure S2A–C). Among these three clones, CTCF bound peaks were evenly distributed in promoters (21.4–30.4%), introns (30.8–35.32%), and distal intergenic regions (33.23–37.66%) (Supplementary Figure S2D and G). Global reduction of CTCF-binding affinity and minimal CTCF retention at certain loci were evident in all three clones following 48 h of IAA treatment (Figure 2A and B; Supplementary Figure S2A–C, Supplementary Table S4).

Given the variation of peak numbers called from different clones, a more stringent analysis of the CTCF-binding signature was conducted by combining peaks called between the three replicates and using only those peaks containing CTCF target sites (CTSes) as well as high confidence reproducibility (see method of ‘Analysis of Cut&Run data’). The ChIPseeker analysis tool (53) was used to detect bias in genomic distribution of CTSes upon IAA treatment for 48 h. From the combined analysis, there were 13, 287 total CTSes assigned to three categories; reduced (6394), gained (153) and retained (6740). Among the reduced CTCF-binding peaks, 31.48% were located at promoter regions within 1 kb of a transcription start site (TSS), and 30.84% were located at distal intergenic regions. In contrast, retained peaks were mostly located at promoters within 1 kb distance of a TSS (75.77%), with less retention at distal intergenic regions (10.98%). Gained CTCF binding peaks shared a similar pattern to retained peaks (75.82% at promoters within 1 kb distance to TSS and 9.8% at distal intergenic regions) (Figure 2C and Supplementary Table S4).

Previously (59), it was observed that CTCF and BORIS (CTCFL) could co-occupy a specific subset of regulatory elements consisting of clustered CTCF-binding motifs (termed 2XCTSes) mainly located at active promoters and enhancers. To separate CTCF peaks with either 1XCTSes or 2XCTSes, we identified the number of CTCF motifs in the SEM genome located within 40-bp of each other and grouped them by counts. For instance, 1XCTSes indicated one CTCF-binding motif with no others counted within 40- bp, while 2XCTSes have 2 CTCF motifs and 3XCTSes have ≥ 3 CTCF motifs. Next, we assigned CTCF peaks identified by Cut&Run to these three groups. Overall, we observed peak density of 2XCTSes and 3XCTSes was generally stronger than 1XCTSes. Upon CTCF loss, the CTCF-binding affinity against 2XCTSes and 3XCTSes was more significantly reduced than 1XCTSes as indicated by the log_2_fold change (CTCF^AID+IAA/-IAA^) (Figure 2D and E, *P* = 2.2e-16, Wilcox test). However, there is no significant difference between loss of binding between 2XCTSes and 3XCTSes (Figure 2E, *P* = 0.51, Wilcox test).

### CTCF depletion reduced global TAD insulation and intra-TAD chromatin loops

**Figure 3.**
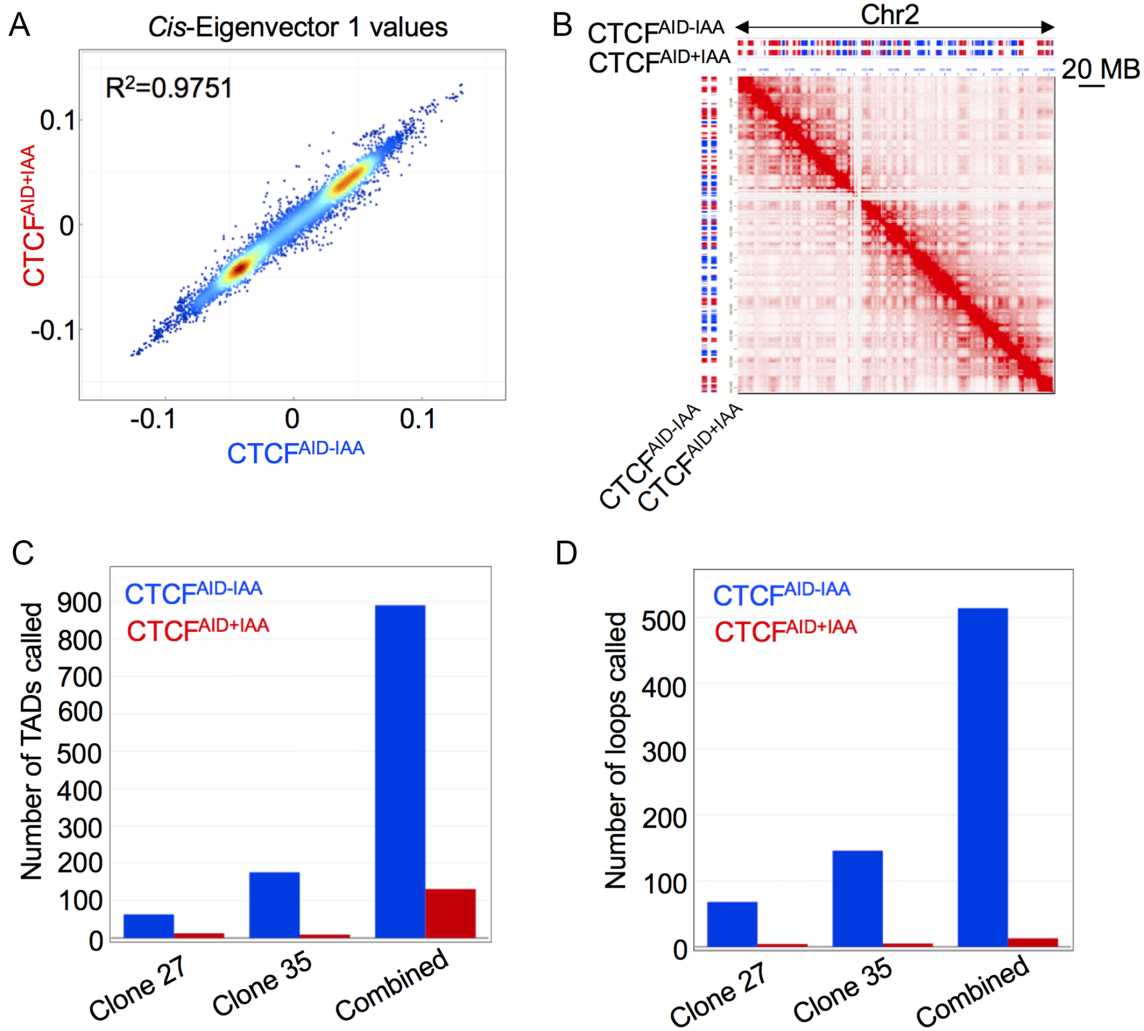
CTCF depletion reduced global TAD insulation and intra-TAD chromatin loops. (**A**) Chromatin compartments evaluated by *cis*-Eigenvector 1 values were not significantly affected genome wide by CTCF protein depletion following 48 h IAA treatment. (**B**) A representative snapshot of chromatin compartment integrity of the entire chromosome 2 was shown in cells from Hi-C data combined from clones 27 and 35 without IAA treatment and following 48 h of IAA treatment. Red bars indicated compartment (A); blue bars indicated compartment (B). Scale bar indicated 20 MB. (**C**) Number of TADs called from clone 27 and 35 were shown in cells with or without 48 h IAA treatment. Reproducibility of Hi-C data from each biological replicate was high enabling compilation of raw Hi-C data for recalling of TADs (combined). (**D**) Number of intra-TAD DNA loops called from clone 27 and 35 were shown in cells with or without 48 h IAA treatment. Reproducibility of Hi-C data from each biological replicate was high enabling compilation of raw Hi-C data for recalling of intra-TAD loops (combined).

To explore global chromatin architecture changes in response to CTCF loss, we utilized *in situ* Hi-C to quantify the genome-wide DNA contacts based on proximity ligation and next-generation sequencing in the SEM clones 27 and 35 after 48 h IAA treatment achieving ∼20 kb resolution. As a quality control, we detected the translocation breakpoint of AFF1/MLL fusion gene, which was initially identified as a driver mutation during leukemogenesis of SEM cells (Supplementary Figure S3A–B). We found that high-order chromosome folding at the resolution of compartments was normally maintained after 48 h of CTCF depletion in SEM cells (*R*^2^ = 0.9751) (Figure 3A and B), which was also supported by other cellular models (29). Next, based on the sequencing depth and resolution of our Hi-C data, we called 63 and 176 TADs in untreated clone 27 and 35, respectively. However, after CTCF depletion 12 and 9 TADs remained, correspondingly. Reproducibility of Hi-C data from each biological replicate was consistently high (Supplementary Figure S3C) enabling compilation of Hi-C data for analysis. When we combined raw Hi-C data collected from both clones and re-called TADs, 890 TADs were discovered in untreated cells and 131 TADs in cells treated with IAA for 48 h (Figure 3C). Additionally, quantification and comparison of the impact of CTCF loss on DNA loops within TAD boundaries showed a 97.5% decrease in intra-TAD looping following 48 h of IAA treatment (Figure 3D). These data suggest that CTCF is essential for maintenance of intra-TAD DNA interactions and delineating TAD boundaries, but dispensable for higher order compartment integrity.

### The global transcriptome is minimally altered upon acute depletion of CTCF

**Figure 4.**
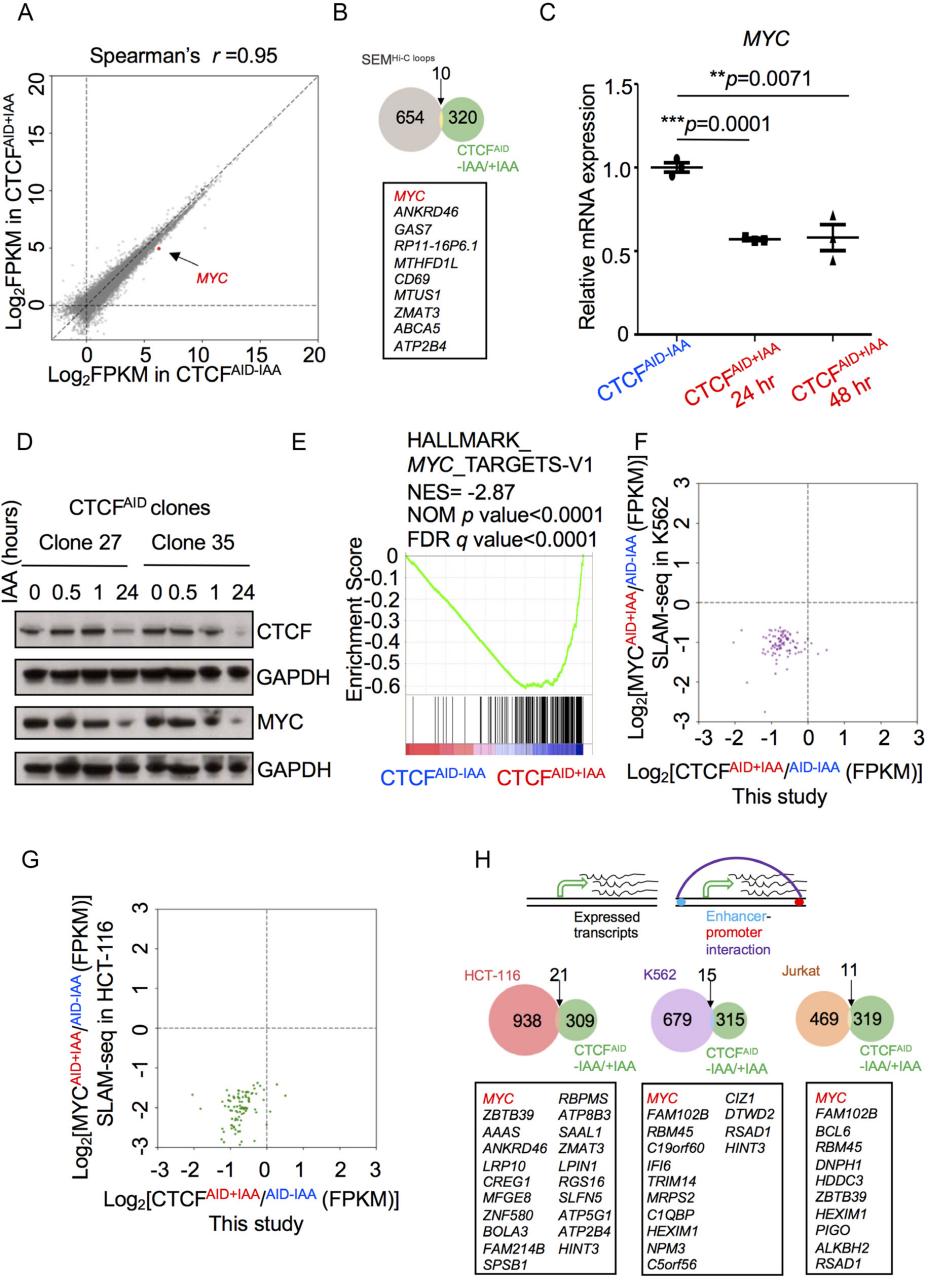
The oncogene *MYC* was directly susceptible to CTCF loss. (**A**) RNA-seq data were illustrated as log_2_ (normalized numbers of FPKM) averaged from three clones (clone 27, 35 and 42) with or without IAA treatment for 48 h. *MYC* was highlighted in red. (**B**) The Venn-diagram illustrated the overlapped gene number (yellow) between genes confirmed by Hi-C to have disrupted intra-TAD looping after CTCF depletion (gray) and genes identified as differentially expressed [Ilog_2_fold change (CTCF^AID-IAA/+IAA^)I≥1, adjust *P* ≤ 0.05 and FPKM≥1] following CTCF depletion (green) for 48 h. (**C**) Q-PCR of *MYC* confirmed the reduction of transcription in response to CTCF depletion for 48 h from three biological replicates; clone 27, 35 and 42 (*N* = 3). (**D**) Clones 27 and 35 were treated with IAA for 0.5, 1 and 24 h and collected for immunoblotting using specific antibodies against CTCF and MYC. GAPDH was used as loading control. (**E**) GSEA analysis was performed using fold change of all genes collected from RNA-seq and DESeq2 analysis by comparing CTCF^AID-IAA^ versus CTCF^AID+IAA^, which revealed the top rank concordance of CTCF depletion with the differential expression of *MYC* target genes. (**F**) The gene expression level of the top 100 downregulated *MYC* target genes discovered by SLAM-seq (61) in MYC^AID^ knock-in K562 cells was plotted to illustrate concordant downregulation in SEM CTCF^AID^ cells after IAA treatment for 48 h. Six genes were not shown because of FPKM≤1. (**G**) The gene expression level of the top 100 downregulated *MYC* target genes discovered by SLAM-seq (61) in MYC^AID^ knock-in HCT-116 cells was plotted to illustrate concordant downregulation in SEM CTCF^AID^ cells after IAA treatment for 48 h. (**H**) Transcriptionally active genes with putative CTCF-mediated enhancer-docking in HCT-116 (red circle), K562 (purple circle) and Jurkat cells (orange circle) were identified from a previous study (62). The Venn-diagram demonstrated the number of overlapped genes between the above studies and genes identified as differentially expressed following CTCF depletion (green circle) [Ilog_2_ (fold change CTCF^AID-IAA/+IAA^)I≥1, adjust *P* ≤ 0.05 and FPKM≥1].

To comprehensively evaluate transcription alteration according to global looping disruption in CTCF-depleted cells, we conducted RNA-seq on SEM clones 27, 35 and 42. At a stringent cut-off (log_2_fold change≥1, adjust *P* ≤ 0.05, FPKM≥1), we identified 330 genes that were differentially expressed between CTCF wild-type (CTCF^AID-IAA^) versus depleted cells (CTCF^AID+IAA^). Overall, the global transcriptome profiling between CTCF^AID-IAA^ and CTCF^AID+IAA^ was highly correlated (Spearman’s *r* = 0.95), suggesting acute depletion of CTCF in the miniAID-mClover3 knock-in SEM clones did not significantly impact global transcription at an early time point (Figure 4A). As seen by Q-PCR, RNA-seq confirmed CTCF expression increased following IAA treatment (Supplementary Figure S5A). Although we observed a significant disruption to intra-TAD interactions and TAD boundary maintenance, transcriptional changes to genes located near abrogated interactions and disrupted TAD boundaries were mostly unaltered. When we compared our transcriptome data with 664 genes confirmed by Hi-C to have altered DNA looping following IAA treatment for 48 h, we only identified 10 genes with overlapping differential expression and abrogated looping (Figure 4B). These data suggest that while CTCF is necessary for many intra-TAD interactions, early loss of CTCF has a limited direct impact on transcription.

GSEA revealed the significant concordance of CTCF depletion with upregulation of the apoptosis pathway, while unsupervised hierarchical transcriptome analysis demonstrated the upregulation of apoptosis associated genes 48 h post IAA treatment (Supplementary Figure S9A and B). Upregulation of the apoptosis pathway upon CTCF depletion supported our observation that loss of CTCF in SEM cells resulted in time-dependent cell death. When IAA was removed from the culture medium, CTCF protein level was restored and cell viability was rescued (data not shown).

### The oncogene *MYC* was most directly susceptible to CTCF loss

Notably, RNA-seq identified the oncogene *MYC* among the genes significantly downregulated by CTCF loss in SEM cells (Figure 4A; Supplementary Figure S4A–F). Global looping analysis additionally confirmed looped DNA interactions between *MYC* and its distal enhancers and TAD boundaries were significantly disrupted following IAA treatment (Supplementary Table S3). Q-PCR confirmed the notable reduction of *MYC* upon CTCF loss following 48 h of IAA treatment in SEM (Figure 4C), and immunoblotting validated the decrease of MYC and CTCF 24 h post IAA treatment (Figure 4D). Additionally, *MYC* expression was completely restored after IAA removal for 48 h (Supplementary Figure S9C). Among the genes located in the same TAD boundary with *MYC*, we observed significant transcriptional changes for the non-coding RNA *PVT1* (Supplementary Figure S5B), which has been identified to share and compete for enhancer activity with *MYC* (60). Other genes in the *MYC* TAD including *GSDMC, TMEM75* and *FAM84B* also appeared differentially regulated by altered CTCF-dependent higher order structure. However, the expression level of these genes was low resulting in poor reproducibility of transcription variation between the replicates (Supplementary Figure S5C–F). The transcription level of other B-ALL associated major oncogenes including *MYB, RUNX1, EBF1, KRAS, NRAS* and *MEF2C* remained unchanged (Supplementary Figure S6A–F).

GSEA revealed the unique and significant concordance of CTCF depletion with downregulation of *MYC* target gene signatures (Figure 4E). Although MYC could potentially regulate thousands of target genes, a recent study by Muhar *et al.* using nascent mRNA sequencing by SLAM-seq identified only 100 direct targets downregulated upon *MYC* loss (61). When we compared these 100 genes from SLAM-seq to our RNA-seq dataset, 92 out of 100 genes showed decreased expression in CTCF depleted cells (Figure 4F and G; Supplementary Figure S7A–F). To address whether the ‘*MYC*-signature’ was unique upon CTCF loss in B-ALL SEM cells, we performed RNA-seq on two well-characterized immortal HUDEP-2 CTCF^AID^ knock-in clones (C3 and C16) (Supplementary Figure S8A–D). In total, 1216 genes were differentially expressed (log_2_fold change≥1, adjust *P* ≤ 0.05) between CTCF wild-type (CTCF^AID-IAA^) versus depleted cells (CTCF^AID+IAA^), with expression recovered in most genes when IAA was removed from the culture medium (Supplementary Figure S8E). However, *MYC* expression was minimally altered upon CTCF depletion (Supplementary Figure S8F). Additionally, MYC downstream targets remained unaffected. The only pathway modestly enriched from GSEA was hedgehog signaling. Collectively, these data support that *MYC* and its downstream targets were primarily affected by acute loss of CTCF in the B-ALL cell line SEM, and that CTCF affected transcriptional regulation in a cell type-specific manner.

Schuijers *et al.* identified a conserved CTCF-binding motif residing 2.5 kb upstream of the *MYC* promoter that provides an enhancer-docking site facilitating its interaction with the downstream *MYC* promoter (62). They go on to postulate that hundreds of putative enhancer-docking sites may be defined by CTCF binding within 2.5 kb of promoters of actively transcribed genes in HCT-116, K562 and Jurkat human cancer cell lines (63,64). When we analyzed our global looping analysis following CTCF depletion to identify disrupted interactions at putative enhancer-docking sites in HCT-116, K562 and Jurkat cells, we only observed abrogated looping in 15, 9 and 8 interactions, respectively. In addition, when we compared the 330 genes we identified as differentially expressed in response to CTCF depletion in SEM cells with the genes classified to contain putative enhancer-docking site interactions in HCT-116, K562 and Jurkat cells, we only identified 21, 15 and 11 overlapped genes, respectively. When we raised the cutoff of mRNA expression (FPKM≥10), the only shared gene between all cohorts was *MYC* (Figure 4H).

### CTCF regulated *MYC* through a long-distance chromatin interaction in SEM cells

**Figure 5.**
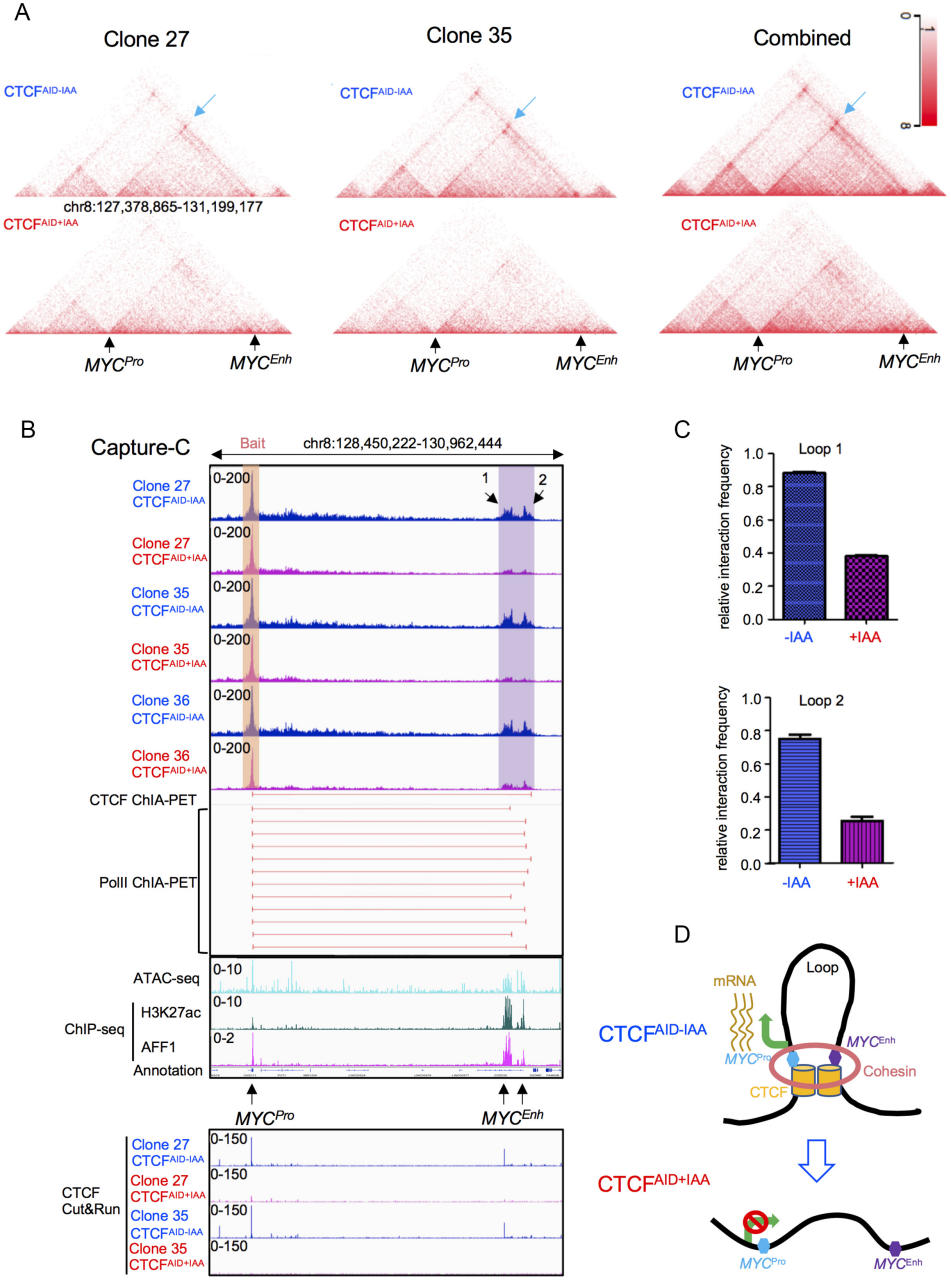
CTCF regulates *MYC* through a long-distance chromatin interaction. (**A**) Snapshots of 12.6 Mb of Hi-C data at 10 kb resolution were shown from CTCF^AID^ SEM clone 27, 35 and Hi-C data combined from clones 27 and 35 without IAA treatment and following 48 h of IAA treatment. DNA interactions between *MYC* and the distal enhancers were highlighted by blue arrows in CTCF^AID-IAA^ samples. (**B**) Next-generation Capture-C was performed on clones 27, 35, and the heterozygous knock-in clone 36 with and without 48 h IAA treatment using specific probes that hybridized to the *MYC* promoter (shown by orange box). The interaction profiles are shown at the indicated viewpoint in the hg19 human genome browser. Interactions to the distal enhancer cluster observed using the *MYC* probes are indicated by the highlighted purple box. Capture-C data were aligned with ATAC-seq, H3K27ac and AFF1 ChIP-seq tracks identified in previous studies (GSE129066, GSE76783 and GSE89485) to specify the functional regulatory elements. CTCF Cut&Run tracks were provided for clones 27 and 35 with or without 48 h IAA treatment. CTCF and PolII ChIA-PET data were obtained from K562 cells from a previous study (GSE39495). (**C**) ‘Relative interaction frequency’ was calculated by the quantification of Capture-C interaction reads from the *MYC* promoter to distal enhancer 1 (arrow 1 = Loop1) and 2 (arrow 2 = Loop 2). (*N* = 2). (**D**) Schematic diagram summary of the CTCF/MYC regulation axis in SEM cells.

MYC overexpression has been detected in 50–60% of human cancers and plays an essential role in cancer initiation and maintenance through multiple molecular mechanisms including gene amplification, aberrant transcription activation and enhancer hijacking. Previous studies have reported the *MYC* promoter is physically recruited to a variety of proximal and distal enhancers to enforce its high expression in different human cancer cells (60,62,65–70). The *MYC* TAD contains many conserved CTCF-binding sites in human cancer and normal somatic cell lines (Supplementary Figure S10). We hypothesized that *MYC* downregulation observed in CTCF-depleted cells was a result of a disrupted CTCF-dependent enhancer–promoter interaction. The Hi-C data collected from SEM clone 27 and 35 indicated dramatic loss of contacts from *MYC* to distal regions in cells treated for 48 h with IAA (Figure 5A). To more clearly identify abrogated interactions, next-generation Capture-C was performed on clones 27, 35 and the mono-allelic knock-in clone 36 with or without IAA treatment using specific DNA probes that hybridize to the *MYC* promoter. We detected frequent interactions between the *MYC* promoter and a distal enhancer cluster residing ∼1.8 Mb downstream of the promoter in the absence of IAA treatment in all three clones. However, the interactions between the distal enhancer cluster and *MYC* promoter were notably disrupted in clone 27 and 35 after IAA treatment, and partially maintained in the heterozygous knock-in clone 36 (Figure 5B and C) indicating CTCF was required to facilitate this specific enhancer–promoter interaction. The distal interacting region has been well supported by previous work to be an enhancer cluster that interacts with the *MYC* promoter. ChIA-PET studies in various cell lines have captured the looped interaction of the *MYC* promoter to the distal enhancer cluster (54). Additionally, the distal enhancer region exhibits hallmark enhancer features including open chromatin status as shown by ATAC-seq (unpublished, GSE129066), and H3K27ac binding as seen by ChIP-seq (71). The enhancer cluster also demonstrated high affinity binding for AFF1 (72). CTCF Cut&Run showed reduced CTCF-binding affinity at the *MYC* promoter and the distal enhancer following 48 h IAA treatment in SEM clones 27 and 35, further supporting *MYC* expression in SEM cells is regulated by a CTCF mediated long-distance chromatin interaction between the distal enhancer and the *MYC* promoter (Figure 5B and D).

*MYC* enhancer–promoter interactions identified in previous works including the enhancer-docking site located 2.5 kb away from the promoter were not detected in our Capture-C analysis (60,62,67,69,73–76). Additionally, Cut&Run assay showed strong CTCF binding at the *MYC* promoter and distal enhancer in untreated SEM clones, with weaker occupancy observed more proximal to the promoter. We conclude that residual expression of *MYC* after IAA treatment might be from basal promoter activity or interactions of the *MYC* promoter with proximal enhancers beyond the detection resolution of Capture-C.

Long non-coding RNA *PVT1* residing ∼58 kb away from *MYC* was also downregulated upon CTCF loss in B-ALL cells. Howard Chang’s group recently showed that targeted CRISPR interference at the *PVT1* promoter enhanced breast cancer cell competition and growth *in vivo* due to the competition between *PVT1* and *MYC* for engagement with four intragenic enhancers in the *PVT1* locus (60). Since the physical distance between *MYC* and *PVT1* is small, juxtaposition between *MYC* and its distal enhancer might also facilitate the interaction and regulation of this enhancer to *PVT1*. However, in our Capture-C data we did not detect any interactions between the *MYC* promoter and *PVT1* or other alternative enhancers when CTCF was depleted. Therefore, *PVT1* downregulation in CTCF depleted cells could be caused by loss of TAD integrity.

## DISCUSSION

CTCF ChIP-seq data collected from different human cancer and normal somatic cells have identified thousands of CTCF-binding sites (40 000–80 000) across the genome with most CTCF-binding sites conserved in TAD boundaries. However, current characterization of chromatin loops discovered by high-resolution Hi-C, Hi-ChIP and ChIA-PET has to date only identified ∼7000 CTCF-mediated loops (77–80) suggesting that most -binding sites are not involved in facilitating DNA interactions, but in other roles such as direct transcriptional regulation. However, we observed that acute depletion of CTCF in SEM and HUDEP-2 cells did not lead to severe genome-wide transcriptional dysregulation. Notably, RNA-seq analysis showed markedly different effects on global transcription in SEM and HUDEP-2 cell lines, again highlighting CTCF functions in a cell type-specific manner to regulate gene expression.

In B-ALL SEM cells, depletion of CTCF significantly disrupted TAD boundaries and intra-TAD DNA loops but had minimal effects on global transcription following 48 h of CTCF depletion. Surprisingly, even some genes located on or near disrupted TAD boundaries did not show transcriptional changes following CTCF depletion suggesting dramatic changes to chromatin architecture may not have an immediate impact on global transcription, and that transcriptional changes secondary to architectural disruption could be observed under continuous depletion of CTCF. However, CTCF depletion was lethal in B-ALL cells, limiting the scope of the experiment. Our findings supported the work by Nora *et al.* that showed loss of Ctcf by the AID system in mouse embryonic stem cells did not abrogate genomic organization on the larger, compartmental scale, but did disrupt TAD insulation and looping between boundaries. While Nora *et al.* carried out IAA treatment for 4 days showing a 10-fold increase in differentially expressed genes over time, we chose to analyze transcriptional changes at an earlier time point to identify genes directly affected by CTCF loss (29). We suspect continued depletion of CTCF over time would result in an accumulation of secondary transcriptional changes in response to dysregulation of genes impacted by early CTCF loss, as well as transcriptional changes brought about by apoptosis signaling.

The *MYC* promoter has been reported to loop to a spectrum of distal and proximal enhancers in different cancers to initiate and maintain tumorigenesis (62,65–67,70,75,76,81). In SEM cells, we showed *MYC* was specifically looped to an enhancer cluster that also resided on the right TAD neighborhood boundary. We hypothesize the transcriptional susceptibility of *MYC* to CTCF regulation is associated with its unique enhancer location, which demonstrated disrupted looping with the *MYC* promoter upon CTCF loss. Paradoxically, when *MYC* was defined to juxtapose both proximal and distal enhancers in myeloid leukemia K562 and WEHI231 B-cell lymphoma (62), ectopic expression of CTCF led to downregulation of *MYC*, growth retardation and promotion of differentiation into the erythroid lineage (25,26) underscoring the complexity of CTCF’s regulation of *MYC* across tissue types.

Survival data collected from a mixed C57BL6/129 strain of *Ctcf^+/−^* mice suggested that ∼80% of heterozygous animals developed multiple tumors compared to 40% in wild-type littermates at ∼100 weeks old. Although these data indicated Ctcf behaved as a tumor suppressor gene (4), which was also supported by somatic mutation burden of CTCF in a recent human pan-cancer study (82), contradictory results were seen in other models regarding the CTCF/MYC regulation axis (25,26,62). Results from this study suggest in B-ALL CTCF could behave indirectly as an oncogenic driver via its regulation of *MYC*. However, additional studies would be necessary to assess CTCF’s role in B-ALL tumorigenesis.

In summary, we developed a simplified, highly efficient knock-in protocol, CHASE-knock-in, to integrate the miniAID-mClover3 cassette to the endogenous human *CTCF* locus, which allowed for acute auxin-inducible degradation of the CTCF protein in human cells. We provide bona fide evidence that CTCF is an essential factor directly regulating the distal enhancer–promoter interactions of *MYC*, suggesting *MYC* and its downstream targets are uniquely affected by acute loss of CTCF in B-ALL SEM cells. The cell model and results collected from this study will expedite the investigation of CTCF’s impact on transcriptional regulation in cancers and other biological processes.

## DATA AVAILABILITY

ChIP-seq data of human normal cell lines and cancer cell lines were obtained from ENCODE. RNA-seq, Capture-C and Cut&Run raw data generated from this study were deposited to GEO under the accession numbers: GSE120781, GSE121257 and GSE126619. SLAM-seq data were downloaded from GSE111463. Hi-C raw data are available at ProteinPaint portal (https://pecan.stjude.org/proteinpaint) upon request. Enhancer–promoter interaction data of HCT-116, K562 and Jurkat were downloaded from GSE92881. CTCF and PolII ChIA-PET data were obtained from GSE39495.

## Supplementary Material

gkz462_Supplemental_Files
