# Supplementary material for: Acute depletion of CTCF directly affects MYC regulation through loss of enhancer–promoter looping
Source: Nucleic Acids Res. 2019 May 25;47(13):6699–713. doi: 10.1093/nar/gkz462 (PMC6648894; doi:10.1093/nar/gkz462)
Supplement: gkz462_Supplemental_Files [file gkz462_supplemental_files.zip › NAR revision 2019-4-22 SUPPLEMENTARY INFORMATION-v2.pdf]

Acute depletion of CTCF directly affects *MYC* regulation through loss of enhancer-promoter looping

Judith Hyle[1,4,†], Yang Zhang[1,†], Shaela Wright[1,4], Beisi Xu[2], Ying Shao[2], John Easton[2], Liqing Tian[2], Ruopeng Feng[3], Peng Xu[3] and Chunliang Li[1,*]

Departments of [1]Tumor Cell Biology, [2]Computational Biology and [3]Hematology, St. Jude Children's Research Hospital, 262 Danny Thomas Place, Memphis, TN 38105, USA

[4]Howard Hughes Medical Institute, Chevy Chase, MD 20815-6789, USA

*Correspondence: chunliang.li@stjude.org

[†]Equal contributors

SUPPLEMENTARY INFORMATION

Supplementary Figures

Figure. S1

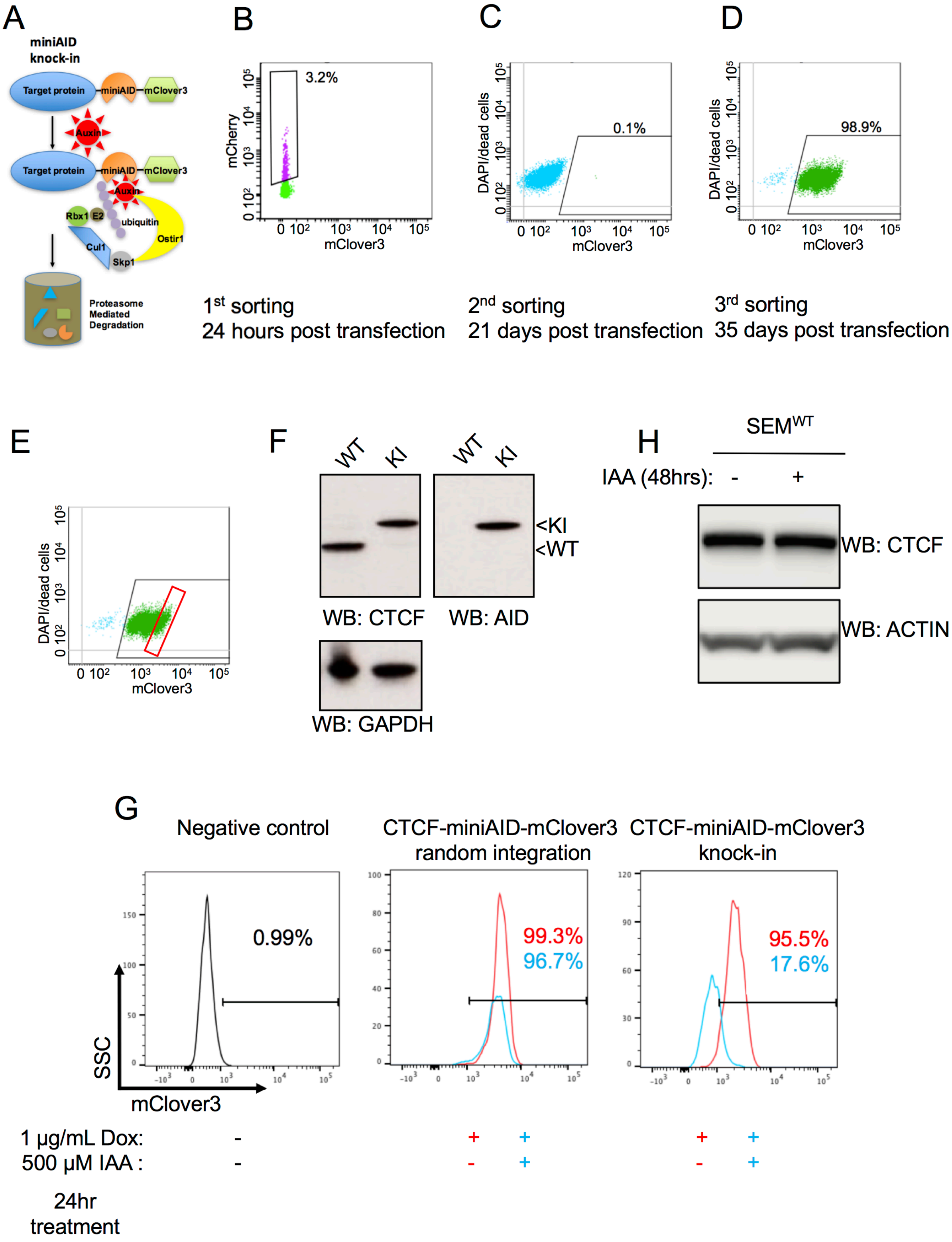
A
miniAID knock-in
Target protein
miniAID
mClover3
Auxin
Rbx1
E2
ubiquitin
Ostir1
Cul1
Skp1
Proteasome Mediated Degradation
B
3.2%
mCherry
mClover3
1st sorting
24 hours post transfection
C
0.1%
DAPI/dead cells
mClover3
2nd sorting
21 days post transfection
D
98.9%
DAPI/dead cells
mClover3
3rd sorting
35 days post transfection
E
DAPI/dead cells
mClover3
F
WT
KI
WT
KI
<KI
<WT
WB: CTCF
WB: AID
WB: GAPDH
H
SEMWT
IAA (48hrs): - +
WB: CTCF
WB: ACTIN
G
Negative control
CTCF-miniAID-mClover3 random integration
CTCF-miniAID-mClover3 knock-in
0.99%
99.3%
96.7%
95.5%
17.6%
SSC
mClover3
1 μg/mL Dox: - + + + +
500 μM IAA : - - + - +
24hr treatment

Supplementary Figure S1. Experimental procedure of CHASE-knock-in protocol.

A. Schematic diagram of auxin-inducible degron model. In the miniAID knock-in system, parental SEM and HUDEP-2 cells were generated by introducing a transgene encoding doxycycline inducible OsTIR1. The miniAID-mClover3 cassette is inserted to the endogenous allele/s in frame with target protein expression in the parental cells. In the presence of doxycycline and auxin, enforced expression of OsTIR1 combines with Skp1/Culin/F-box (SCF) ubiquitin ligase components in the cell to form a functional SCF/OsTIR1 E3 ubiquitin ligase complex that rapidly degrades miniAID-mClover3 fusion proteins in minutes.

B. SEM cells were transfected by electroporation to deliver the CTCF-miniAID-mClover3 vector and CRISPR/Cas9-CTCF-gRNA vector. Twenty-four hours after transfection, cells were sorted for the CRISPR/Cas9-CTCF-gRNA vector mCherry fluorescent marker to enrich the transfected cell population.

C. After sorted cells recovered in culture for up to 3 weeks, a second sort was performed to select cells for successful knock-in by sorting for cells expressing the CTCF-miniAID-mClover3 vector mClover3 fluorescent marker.

D. Two weeks later, a third sort was performed for the mClover3 fluorescent marker. Following the third sort, the population of cells was enriched for stable integration of the miniAID-mClover3 cassette knock-in.

E. CTCF-miniAID-mClover3 knock-in SEM cells were sorted for mClover3. Cells expressing the highest density of fluorescence (red box) were selected to confirm knock-in populations.

F. The high fluorescent density population from “E” (KI) was collected for immunoblotting using antibodies against CTCF and AID. In the CTCF immunoblot, the higher molecular weight band (~35 KD larger than WT CTCF) in the KI cells showed successful knock-in of miniAID-mClover3 to the C-terminus of CTCF. The absence of the WT CTCF band in the KI cells indicated the high-density fluorescent population only expressed the integrated CTCF-miniAID-mClover3 fusion protein. The AID immunoblots confirmed successful integration of the miniAID-mClover3 cassette to the CTCF allele in the KI sample. GAPDH was included as a loading control.

G. Knock-in clone 27 and a random knock-in clone 1 (the miniAID-mClover3 cassette was integrated in a genomic region other than the CTCF locus and demonstrated mCLover3 expression) were treated with doxycycline (1 μg/mL) and IAA (i.e., auxin, 500 μM) for 24 hours to induce degron mediated protein degradation of CTCF. Flow cytometric analysis of mClover3 was performed to quantify the degradation efficiency of the CTCF-miniAID-mClover3 fusion protein upon IAA treatment. Wild-type SEM cells were included as a negative control.

H. Parental wild-type SEM cells were treated with IAA and doxycycline for 48 hours and collected for immunoblotting.

Figure. S2

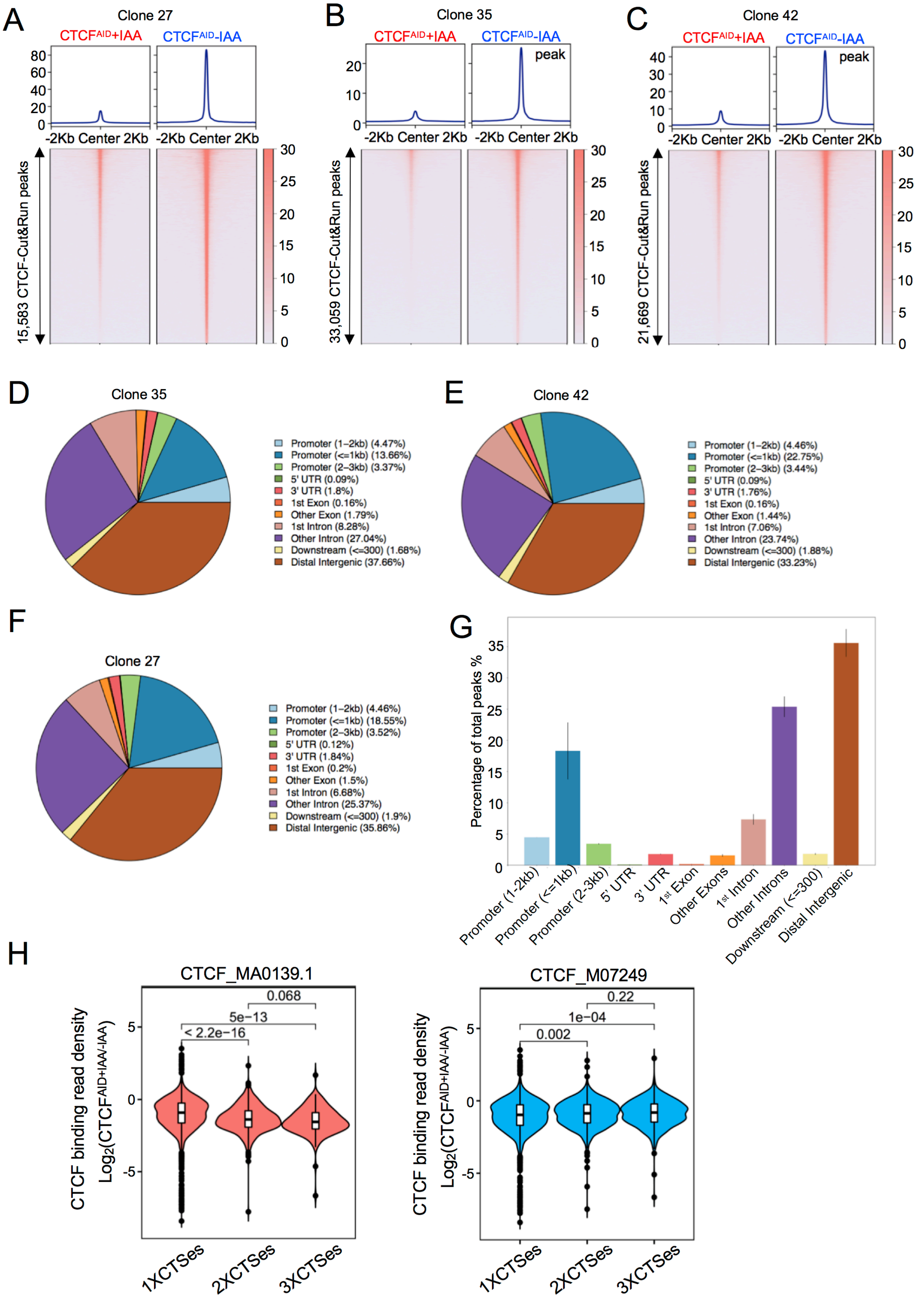
A
Clone 27
CTCF^AID+IAA
CTCF^AID-IAA
-2Kb Center 2Kb
15,583 CTCF-Cut&Run peaks
B
Clone 35
peak
33,059 CTCF-Cut&Run peaks
C
Clone 42
21,669 CTCF-Cut&Run peaks
D
Clone 35
Promoter (1–2kb) (4.47%)
Promoter (<=1kb) (13.66%)
Promoter (2–3kb) (3.37%)
5' UTR (0.09%)
3' UTR (1.8%)
1st Exon (0.16%)
Other Exon (1.79%)
1st Intron (8.28%)
Other Intron (27.04%)
Downstream (<=300) (1.68%)
Distal Intergenic (37.66%)
E
Clone 42
Promoter (1–2kb) (4.46%)
Promoter (<=1kb) (22.75%)
Promoter (2–3kb) (3.44%)
5' UTR (0.09%)
3' UTR (1.76%)
1st Exon (0.16%)
Other Exon (1.44%)
1st Intron (7.06%)
Other Intron (23.74%)
Downstream (<=300) (1.88%)
Distal Intergenic (33.23%)
F
Clone 27
Promoter (1–2kb) (4.46%)
Promoter (<=1kb) (18.55%)
Promoter (2–3kb) (3.52%)
5' UTR (0.12%)
3' UTR (1.84%)
1st Exon (0.2%)
Other Exon (1.5%)
1st Intron (6.68%)
Other Intron (25.37%)
Downstream (<=300) (1.9%)
Distal Intergenic (35.86%)
G
Percentage of total peaks %
Promoter (1-2kb)
Promoter (<=1kb)
Promoter (2-3kb)
5' UTR
3' UTR
1st Exon
Other Exons
1st Intron
Other Introns
Downstream (<=300)
Distal Intergenic
H
CTCF_MA0139.1
CTCF binding read density
Log2(CTCF^AID+IAA/-IAA)
< 2.2e−16
5e−13
0.068
1XCTSes
2XCTSes
3XCTSes
CTCF_M07249
0.002
1e−04
0.22

Supplementary Figure S2. Genomic distribution of CTCF binding peaks

A. Cut&Run assay identified global reduction in CTCF occupancy upon depletion of CTCF following 48 hours IAA treatment ($CTCF^{AID}$+IAA). Peak call analysis was performed in clone 27 by *p*-value cutoff at 1.00e-03.

B. Cut&Run assay identified global reduction in CTCF occupancy upon depletion of CTCF following 48 hours IAA treatment ($CTCF^{AID}$+IAA). Peak call analysis was performed in clone 35 by *p*-value cutoff at 1.00e-03.

C. Cut&Run assay identified global reduction in CTCF occupancy upon depletion of CTCF following 48 hours IAA treatment ($CTCF^{AID}$+IAA). Peak call analysis was performed in clone 42 by *p*-value cutoff at 1.00e-03.

D. Genomic distribution of CTCF binding peaks in clone 35.

E. Genomic distribution of CTCF binding peaks in clone 42.

F. Genomic distribution of CTCF binding peaks in clone 27.

G. Statistical analysis of genomic distribution of CTCF binding peaks collected from three individual clones.

H. Global decrease of CTCF binding affinity by annotating 1XCTSes, 2XCTSes and 3XCTSes with motif scan reference including CTCF_MA0139.1 and CTCF_M07249. P-value was provided among each pairs.

Figure. S3

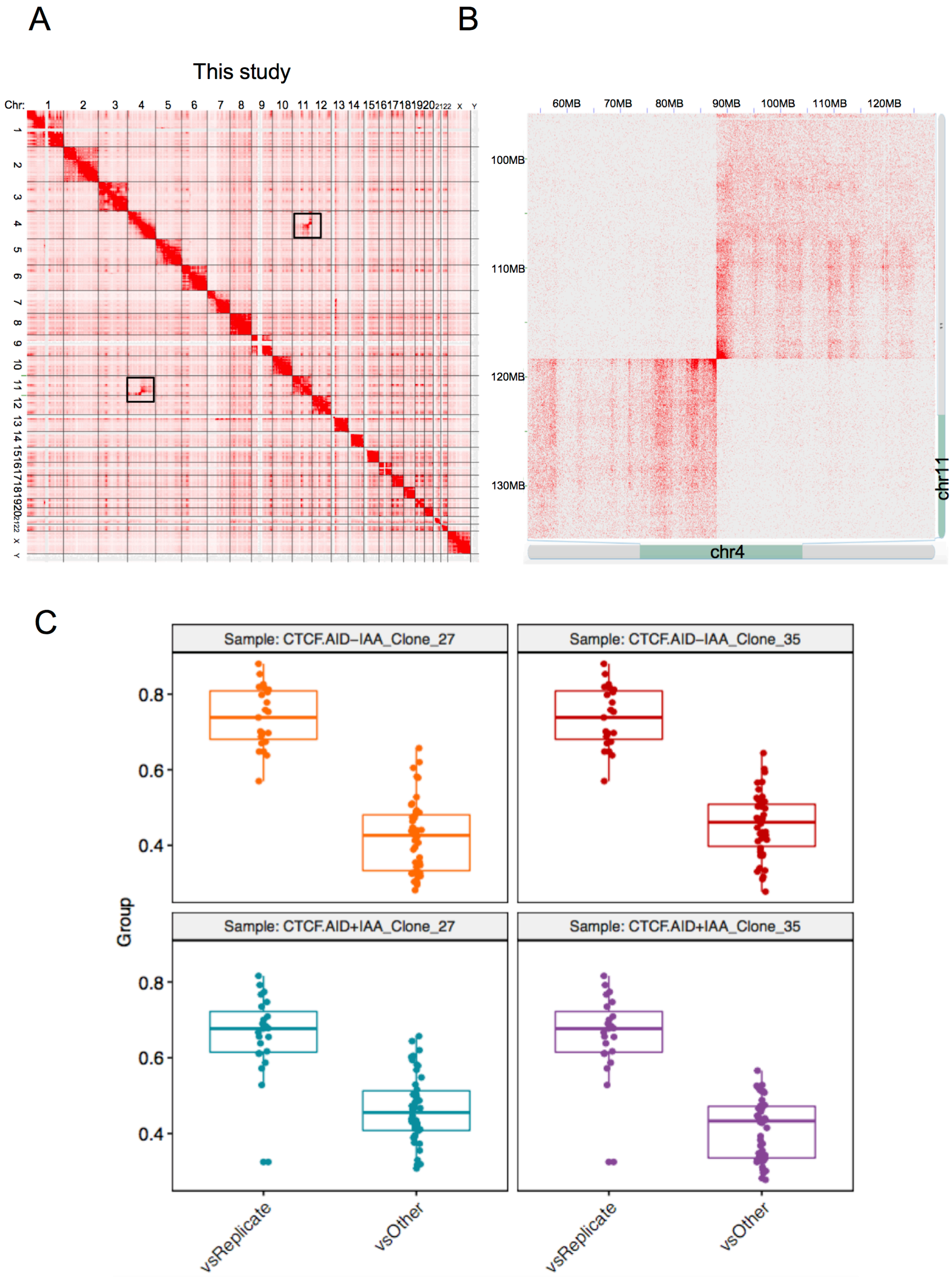
A
This study
Chr:
B
60MB
70MB
80MB
90MB
100MB
110MB
120MB
130MB
chr11
chr4
C
Sample: CTCF.AID−IAA_Clone_27
Sample: CTCF.AID−IAA_Clone_35
Sample: CTCF.AID+IAA_Clone_27
Sample: CTCF.AID+IAA_Clone_35
0.8
0.6
0.4
Group
vsReplicate
vsOther

Supplementary Figure S3. Overview of Hi-C data of CTCF^{AID} parental cells

A. Snapshot of Hi-C data collected from CTCF^{AID} parental SEM cells. Translocation breakpoint of AFF1/MLL (t4,11) was highlighted by black box.

B. Snapshot of the AFF1/MLL translocation breakpoint at higher magnification.

C. Reproducibility analysis of Hi-C data in each biological replicate (N=2). Each dot is a reproducibility score for one chromosome. “vsReplicate” means labeled sample compared to the other replicate (e.g. minus 27 vs 35), “vsOther” means labeled samples compared to other samples that were not replicates (e.g. minus 27 vs plus 27 and minus 27 vs plus 35).

Figure. S4

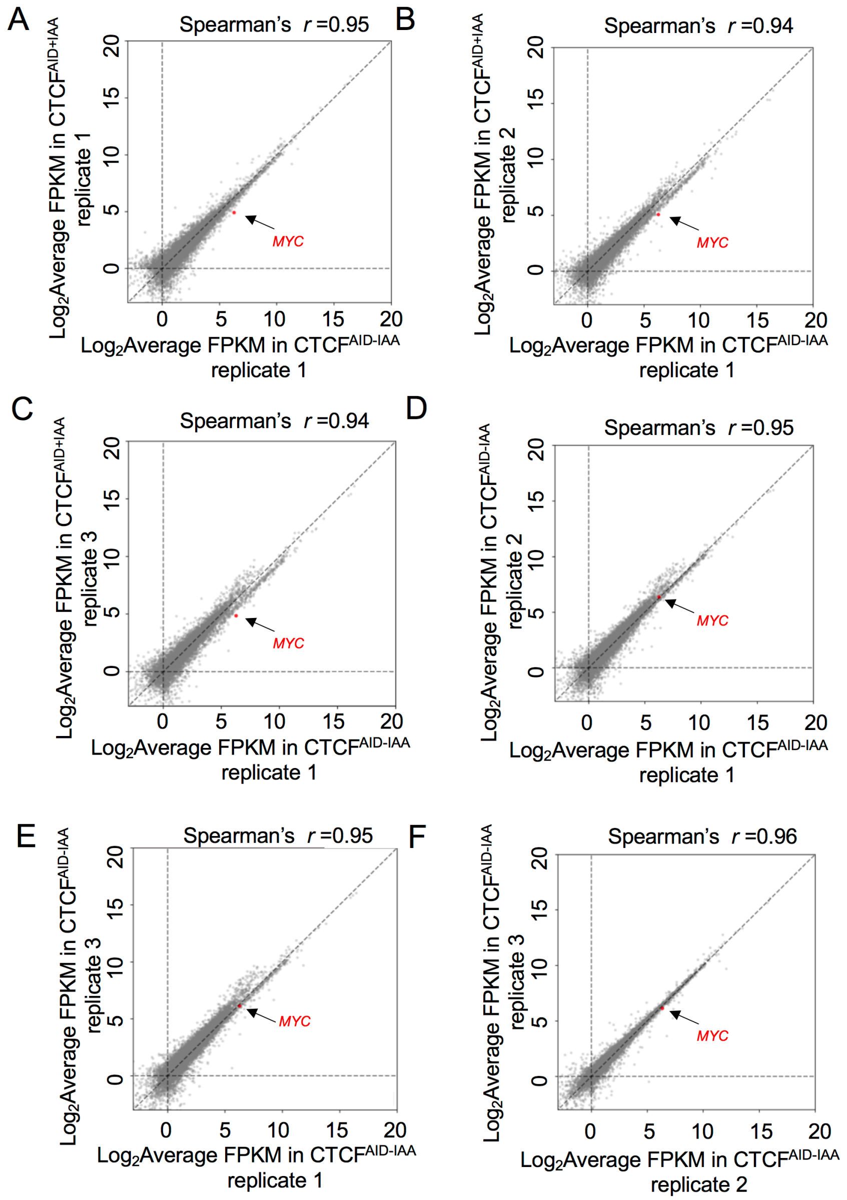

Supplementary Figure S4. Correlation of normalized reads of transcribed genes (FPKM ≥1) between $CTCF^{AID-IAA}$ and $CTCF^{AID+IAA}$ cells.

Three knock-in clones with or without IAA treatment were collected for RNA-seq analysis and plotted to determine Spearman's correlation between each other based on FPKM≥1. *MYC* is highlighted in red and by an arrow.

Figure. S5

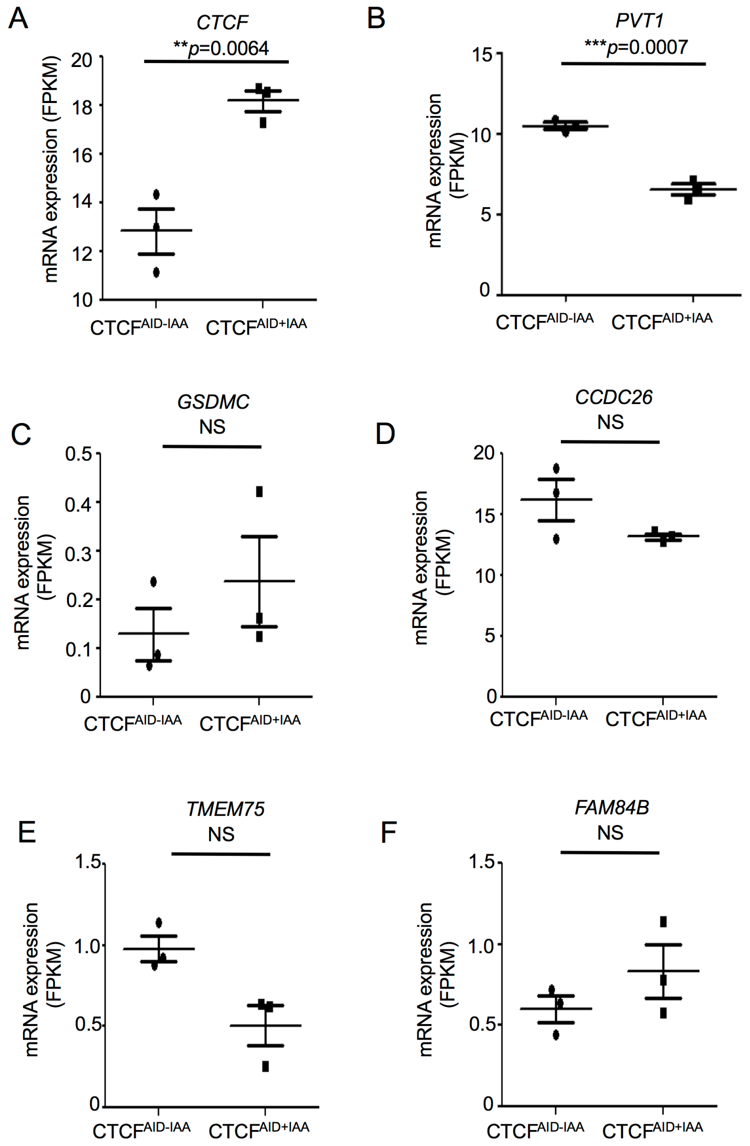

Supplementary Figure S5. Validation of mRNA expression of *CTCF* and other genes residing on the same TAD with *MYC* in response to IAA treatment.

The mRNA level of each gene (FPKM) was analyzed in response to CTCF protein degradation after IAA treatment for 48 hours. Data represent the mean ± SD collected from three independent single cell-derived clones 27, 35, and 42. (N=3)

Figure. S6

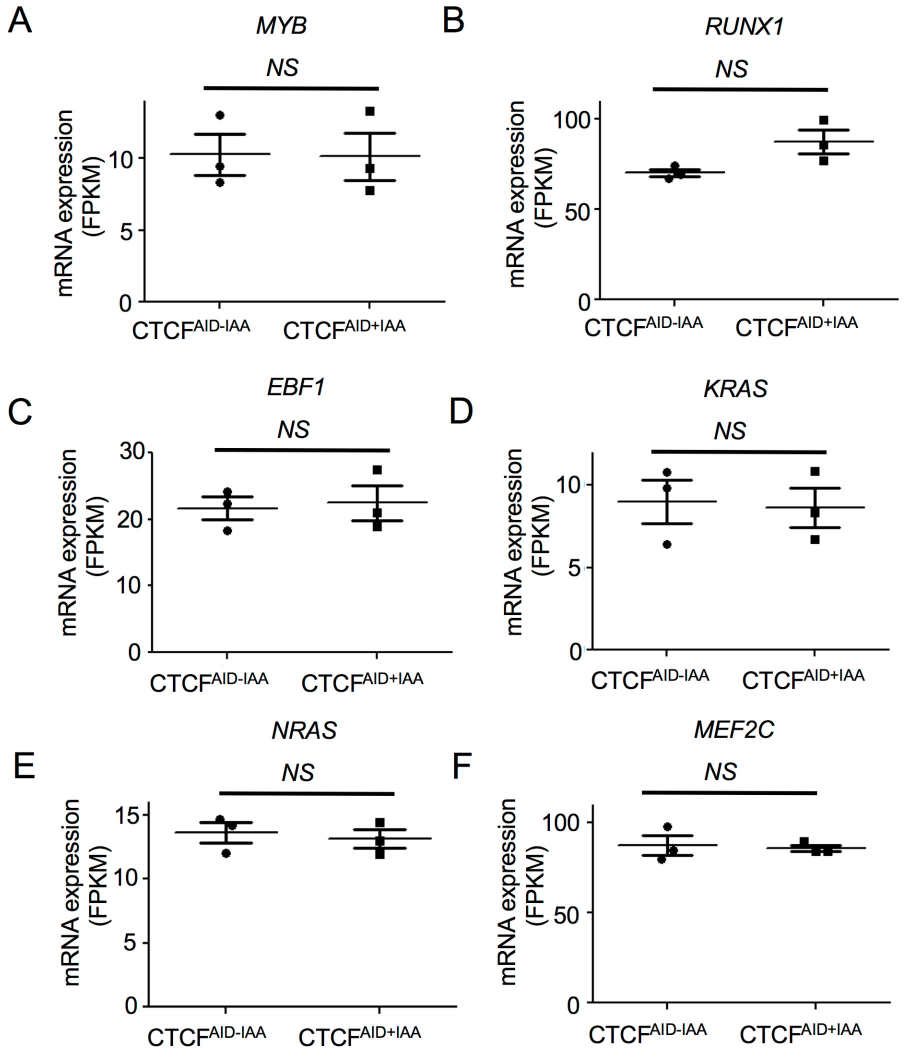

Supplementary Figure S6. Validation of mRNA expression of *CTCF* and other B-ALL associated oncogenes in response to IAA treatment.

The mRNA level of known B-ALL associated oncogenes (FPKM) were analyzed in response to CTCF protein degradation after IAA treatment for 48 hours. Data represent the mean ± SD collected from three independent single cell-derived clones 27, 35, and 42. (N=3)

Figure. S7

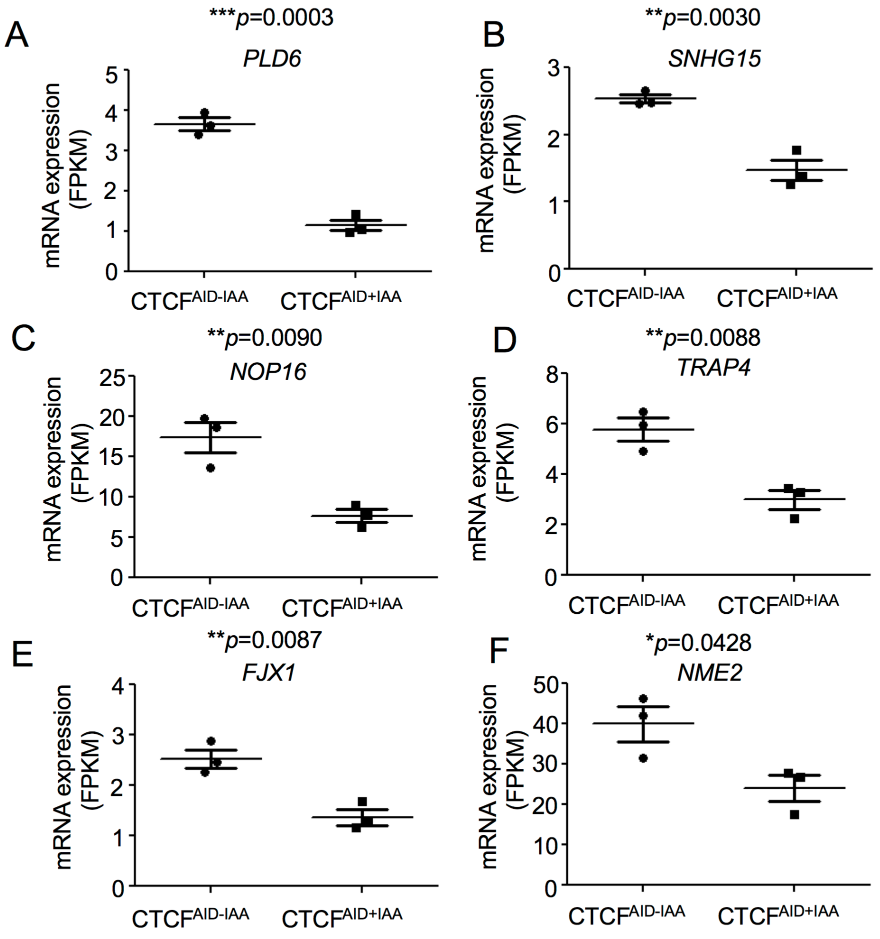

Supplementary Figure S7. Validation of mRNA expression of *MYC* target genes.

The mRNA expression of *MYC* downstream genes is shown by FPKM derived from RNA-seq data. Data represent the mean ± SD collected from three independent single cell-derived clones 27, 35, and 42. (N=3)

Figure. S8

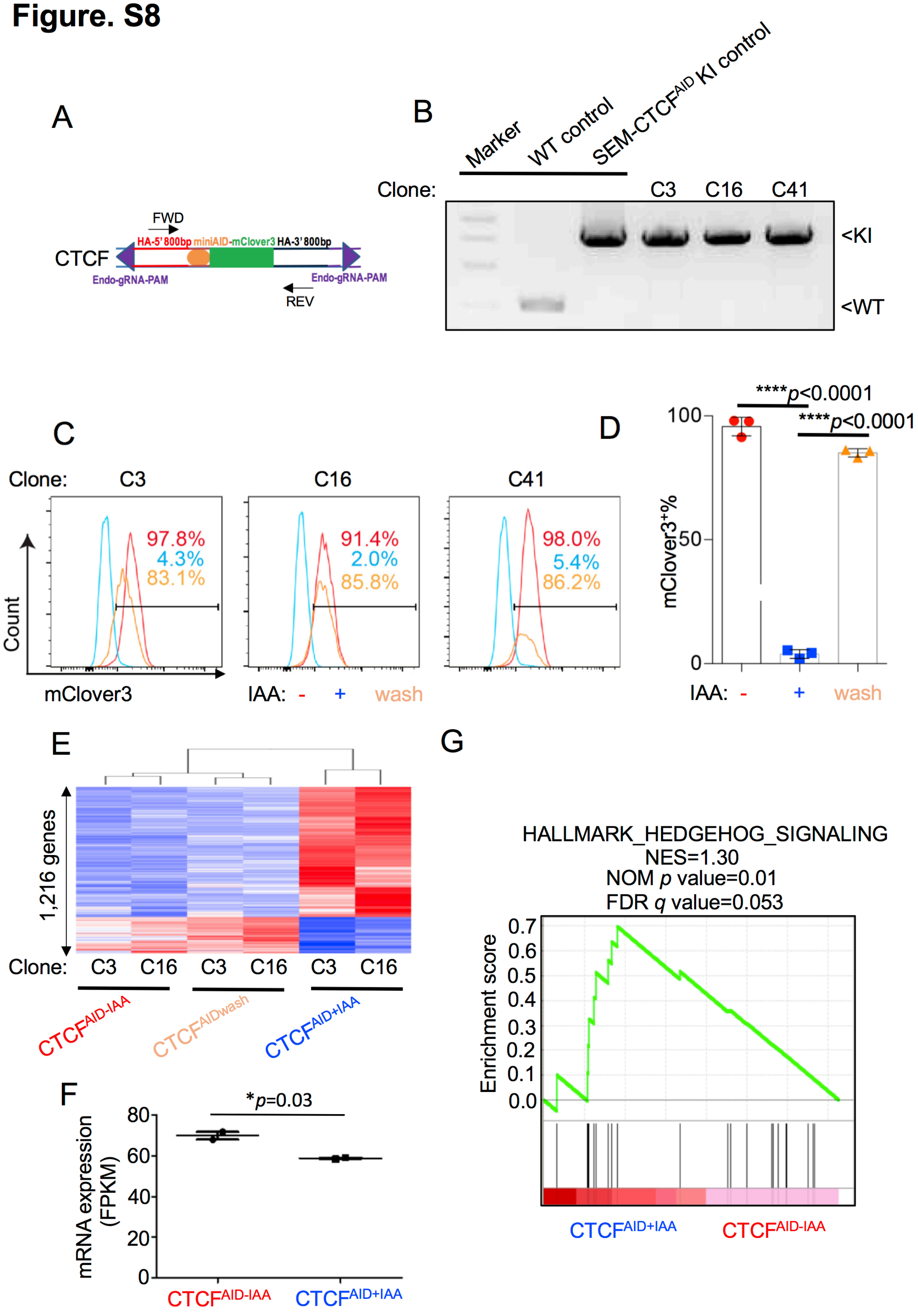

A
FWD
HA-5'800bp miniAID-mClover3 HA-3'800bp
CTCF
Endo-gRNA-PAM
Endo-gRNA-PAM
REV
B
Marker
WT control
SEM-CTCF^AID KI control
Clone:
C3
C16
C41
<KI
<WT
C
Clone:
C3
C16
C41
97.8%
4.3%
83.1%
91.4%
2.0%
85.8%
98.0%
5.4%
86.2%
Count
mClover3
IAA: - + wash
D
****p<0.0001
****p<0.0001
100
50
0
mClover3+%
IAA: - + wash
E
1,216 genes
Clone: C3 C16 C3 C16 C3 C16
CTCF^AID-IAA
CTCF^AIDwash
CTCF^AID+IAA
F
*p=0.03
80
60
40
20
0
mRNA expression (FPKM)
CTCF^AID-IAA
CTCF^AID+IAA
G
HALLMARK_HEDGEHOG_SIGNALING
NES=1.30
NOM p value=0.01
FDR q value=0.053
Enrichment score
0.7
0.6
0.5
0.4
0.3
0.2
0.1
0.0
CTCF^AID+IAA
CTCF^AID-IAA

Supplementary Figure S8. Transcriptome analysis of acute depletion of CTCF in control HUDEP-2 cells.

A. Schematic diagram of genotyping PCR design to identify successful miniAID-mClover3 knock-in events.

B. Knock-in alleles were confirmed by genomic PCR in clones C3, C16, and C41.

C. Three single-cell derived HUDEP-2 clones carrying CTCF-miniAID-mClover3 knock-in cassettes were treated with doxycycline and IAA for 48 hours and harvested for flow cytometric analysis of mClover3. After washing of IAA and doxycycline and culturing for additional 48 hours, restoration of mClover3 was determined by flow cytometric analysis. Red: no IAA treatment; Blue: IAA treatment for 48 hours; Orange: washout for additional 48 hours.

D. Statistical quantification of mClover3 expression in three clones shown in C.

E. Hierarchical analysis of 1,216 differentially expressed genes defined by [$|\log_2$fold change ($CTCF^{AID-IAA/+IAA}$)$|\geq 1$ and adjust $p \leq 0.05$]. Gene expression was normalized and indicated as Z score.

F. FPKM value associated with mRNA expression of *MYC* were collected from RNA-seq data of two HUDEP-2 clones (C3 and C16) and analyzed by t-test. *$p<0.05$.

G. GSEA analysis utilizing RNA-seq data reveals the only significant concordance between CTCF depletion and the expression of hedgehog signaling pathway.

Figure. S9

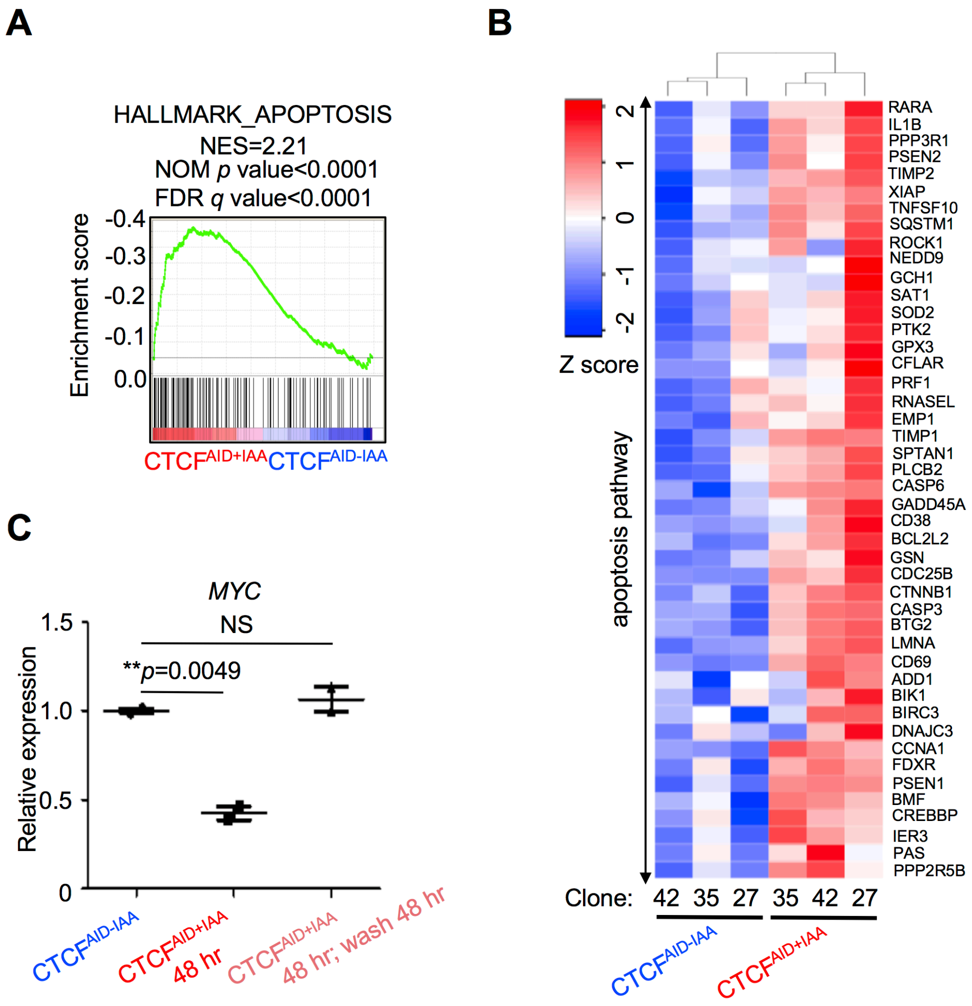

Supplementary Figure S9. Auxin induced CTCF depletion induced cell death and was reversible

A. GSEA analysis from RNA-seq reveals the top rank concordance of CTCF depletion with the activation of the apoptosis signaling pathway.

B. Hierarchical heap map of marker genes involved in apoptosis pathways was shown. Gene expression was normalized and indicated as Z score.

C. Q-PCR of *MYC* confirmed the reduction of transcription level in response to CTCF depletion and restoration after IAA washout. (N=2)

Figure. S10

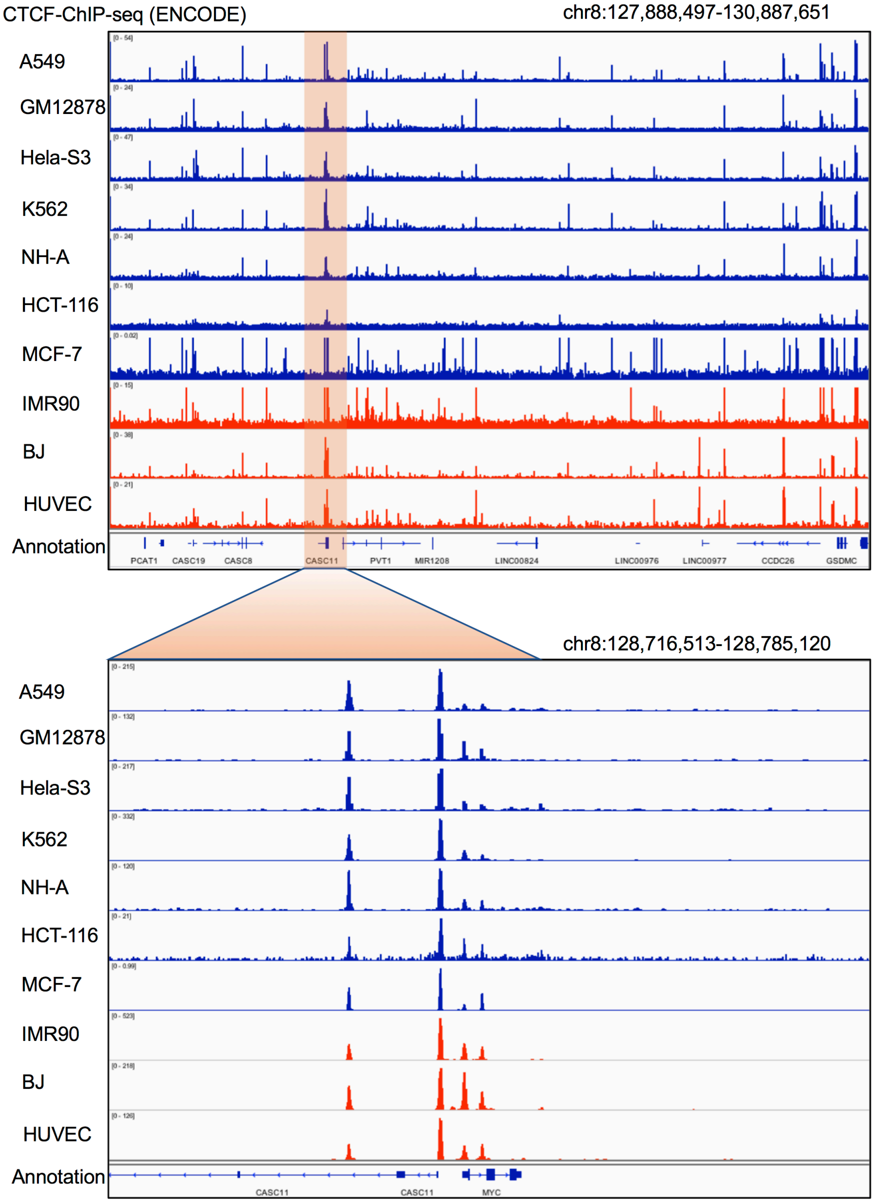

Supplementary Figure S10. Characterization of CTCF binding profiling at *MYC* locus in human cell lines from the ENCODE database.

ChIP-seq tracks of CTCF are shown from various human cancer cell lines (blue) and normal somatic cell lines (red) at the viewpoint of the different viewpoints.

Additional Data Tables (separate files)

Supplementary Table S1. Single-guide RNA sequences, Capture-C oligonucleotides, Q-PCR primers, and cloning primers used for making CHASE-knock-in donor vectors were included.

Supplementary Table S2. FPKM values from transcriptome analysis of SEM $CTCF^{AID}$ with or without 48 hours IAA treatment were included.

Supplementary Table S3. Intra-TAD DNA loop summarization of SEM $CTCF^{AID}$ Hi-C analysis with or without 48 hours IAA treatment was included.

Supplementary Table S4. CTCF Cut&Run peak information associated with Figure 2 and Figure S2.
